# Activating Ferroptosis of M1 Macrophages: A Novel Mechanism of Asiaticoside Encapsuled in GelMA for Anti‐Inflammation in Diabetic Wounds

**DOI:** 10.1002/EXP.20240062

**Published:** 2025-10-31

**Authors:** Shengnan Cui, Sheng Meng, Yong Liu, Shengqiu Chen, Wenzhi Hu, Qilin Huang, Ziqiang Chu, Weicheng Zhong, Liqian Ma, Zhe Li, Yufeng Jiang, Xi Liu, Xiaobing Fu, Cuiping Zhang

**Affiliations:** ^1^ Medical Innovation Research Department PLA General Hospital and PLA Medical College Beijing China; ^2^ Department of Dermatology The Second Affiliated Hospital Shaanxi University of Chinese Medicine Xianyang Shaanxi China; ^3^ Department of Dermatology The Second Affiliated Hospital of Xi'an Jiaotong University Xi'an Shaanxi China; ^4^ Innovation Research Center for Diabetic Foot West China Hospital Sichuan University Chengdu China; ^5^ PLA Key Laboratory of Tissue Repair and Regenerative Medicine and Beijing Key Research Laboratory of Skin Injury Repair and Regeneration Beijing China; ^6^ Research Unit of Trauma Care Tissue Repair and Regeneration Chinese Academy of Medical Sciences Beijing China; ^7^ Burns Unit Concord Hospital University of Sydney Medical School Sydney Australia; ^8^ Department of Tissue Regeneration and Wound Repair PLA General Hospital Beijing China

**Keywords:** asiaticoside, anti‐inflammation, diabetic wounds, ferroptosis, macrophages

## Abstract

Diabetic wounds are characterized by chronic inflammation, partly due to the persistent accumulation of pro‐inflammatory M1 macrophages. Asiaticoside (AS), a triterpenoid extracted from *Centella asiatica*, has known anti‐inflammatory effects in several diseases, but the underlying mechanisms in diabetic wounds are still unclear. This study reveals that AS alleviates inflammation in diabetic wounds by activating ferroptosis of M1 macrophages. In vitro, AS reduces the number of M1 macrophages in a high glucose microenvironment and their secretion of proinflammatory cytokines with concurrent induction of ferroptosis. Further investigation shows that AS‐activated ferroptosis is attributed to the downregulation of ferroportin 1 (FPN1) and ferritinophagy‐induced degradation of ferritin heavy chain 1 (FTH1), which together increase the amount of intracellular free ferrous ions (Fe^2+^). In vivo, AS‐encapsulated gelatin‐methacryloyl hydrogels accelerates diabetic wound healing and shortens the inflammatory period by activating ferroptosis of M1 macrophages with the reduced expression of FPN1 and FTH1. These results suggest a promising AS‐based strategy for treating inflammatory diseases associated with excessive activation of M1 macrophages.

## Graphical abstract

Asiaticoside (AS) has known anti‐inflammatory effects in diabetic wounds, but the underlying mechanisms are still unclear. In this study, we reveal that AS alleviates inflammation in diabetic wounds by activating ferroptosis of M1 macrophages, which is attributed to the downregulation of ferroportin 1 and ferritinophagy‐induced degradation of ferritin heavy chain 1. Furthermore, AS was encapsulated in gelatin‐methacryloyl hydrogels to accelerate diabetic wound healing and shorten the inflammatory period by activating ferroptosis of M1 macrophages in vivo. This study suggests a promising AS‐based strategy for treating inflammatory diseases associated with excessive activation of M1 macrophages.

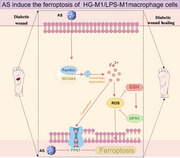

## 1 Introduction

With the surges of diabetes, the incidence of diabetes‐related chronic refractory wounds, such as diabetic foot ulcers (DFUs), has increased significantly [1, 2, 3]. Pathologically, the long‐lasting chronic inflammation is one of the typical features of diabetic wounds and is primarily associated with the excessive activation of inflammatory cells including macrophages [4, 5, 6]. Macrophages play a crucial role in the wound healing process [7]. Under healthy conditions, proinflammatory M1 macrophages are recruited to the wound sites to clear pathogens in the early inflammatory phase. At the end of the inflammatory phase, M1 macrophages differentiate into anti‐inflammatory M2 macrophages to resolve inflammation and promote cell proliferation [8]. However, under diabetic conditions, the transition from M1 to M2 phenotype is hindered significantly [9, 10, 11] and phagocytosis and bactericidal activities are decreased in M1 macrophages [12]. These cues synergistically lead to excessive accumulation of M1 macrophages at diabetic wounds, leading to the long‐lasting inflammation. Numerous anti‐inflammatory strategies have been proposed to accelerate diabetic wound healing. For example, Shen et al. developed melanin@Pt nanoparticles and melanin@AuPt nanoparticles incorporated hydrogels to regulate macrophage polarization, scavenge reactive oxygen species (ROS), and promote tissue repair [13, 14, 15]. Wang et al. designed a series of copper (Cu)‐based and ruthenium (Ru)‐based metalloenzymes to regulate ROS, defeat bacterial infection, and accelerate healing process [16, 17, 18, 19, 20]. However, how to reduce the number of inflammatory M1 macrophages has not been primarily thoroughly.

In China, *Centella Asiatica*, a traditional Chinese herb, has been used to treat wounds for centuries [21, 22]. Asiaticoside (AS), the main bioactive triterpenoid component extracted from *Centella Asiatica*, is widely used in clinical applications in the form of a cream to promote the healing process [23, 24]. AS has multiple biofunctions including promoting angiogenesis and collagen synthesis as well as inhibiting scar formation through the interaction with wound repair cells [25, 26]. Recently, the anti‐inflammation properties of AS have attracted considerable attention. For example, AS could suppress inflammatory responses in acute lung injury and osteosarcoma by regulating the behavior of macrophages [27, 28, 29]. However, the effects of AS on macrophage function in diabetic wounds and the underlying molecular mechanisms remain unclear.

Ferroptosis, a new form of regulatory cell death discovered in the last decade, is characterized by the overload of intracellular free ferrous ions (Fe^2+^), the accumulation of iron‐dependent lipid peroxide, and the deformation of mitochondria [30]. Reportedly, ferroptosis in immune cells plays a crucial role in the development of immune‐associated diseases. For example, neutrophils or CD8^+^ T cells underwent ferroptosis in the tumor microenvironment, ultimately leading to immune escape and rapid proliferation of tumor cells [31]. As a therapeutic target, inducing iron‐dependent ferroptosis has shown promise for alleviating eosinophilic airway inflammation [32]. Similarly, the treatment of LPS‐stimulated microglia and peritoneal macrophages with a ferroptosis activator inhibited the secretion of proinflammatory cytokines. This phenomenon can be attributed to the excessive accumulation of Fe^2+^ and the subsequent overload of ROS in M1 macrophages [33]. However, it is unclear whether AS can attenuate diabetic wound inflammation by targeting ferroptosis of M1 macrophages.

**FIGURE 1:**
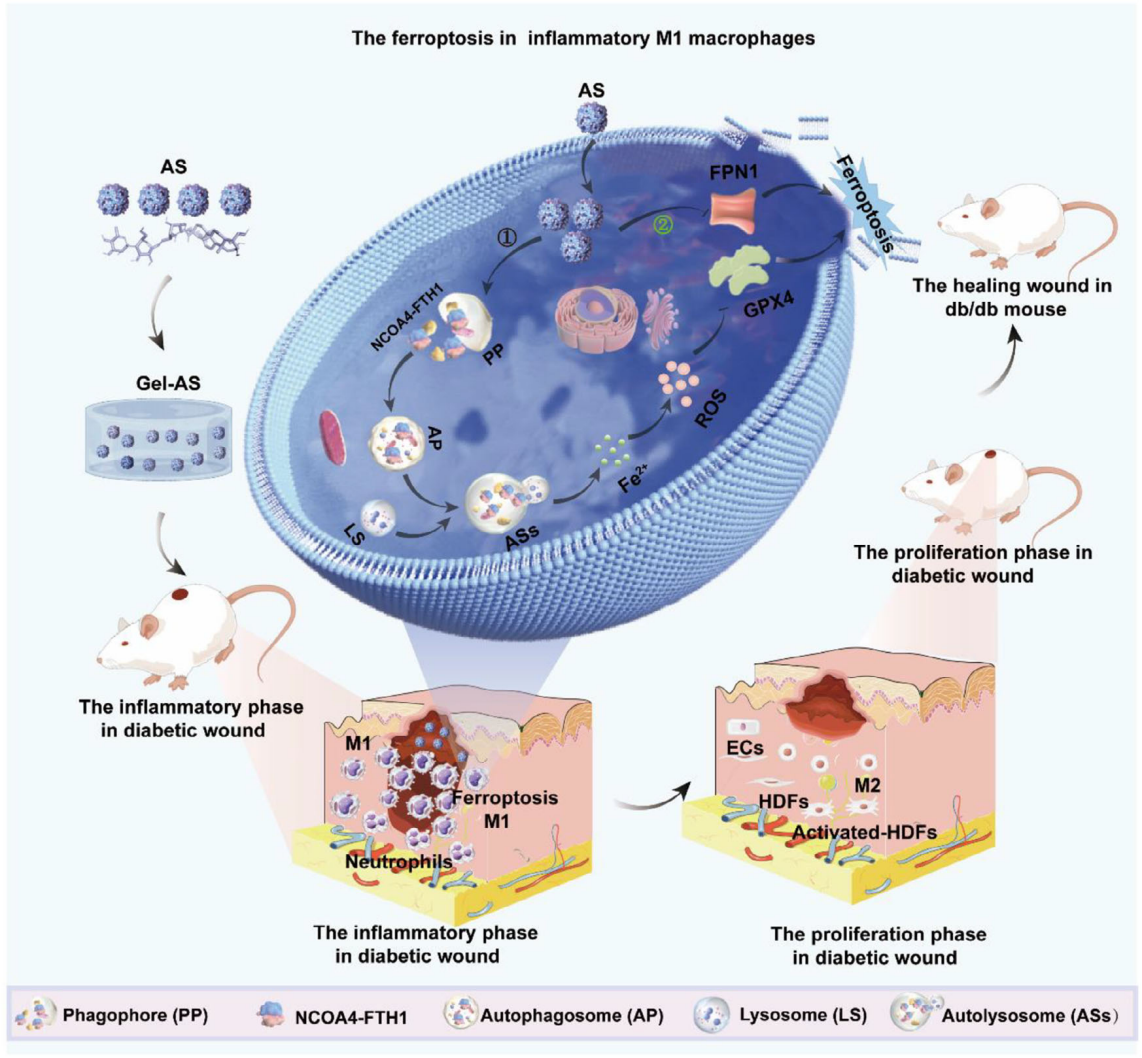
Mechanism illustration of Gel‐AS for anti‐inflammation in diabetic wounds via activating iron metabolism‐dependent ferroptosis in M1 macrophages. **①** represents that AS activates ferritinophagy to degrade FTH1 to release a massive Fe^2+^. **②** represents that AS inhibits the expression of FPN1 to block the transportation of Fe^2+^. The figure was generated under the assist of Figdraw (www.figdraw.com).

Herein, we aspired to explore the underlying molecular mechanisms responsible for the excellent anti‐inflammatory properties of AS on M1 macrophages. First, we observed that AS reduced the number of high glucose (HG)/lipopolysaccharide (LPS)‐M1 macrophages and downregulated the secretion of proinflammatory cytokines in a dose‐dependent manner. Then, in vitro experiments revealed an orchestrated mechanism behind the immunoinhibitory effect of AS on M1 macrophages. Specifically, AS triggered ferroptosis in M1 macrophages by simultaneously activating the ferritinophagy and reducing ferroportin 1 (FPN1) expression (Figure 1). To make AS adaptive for the diabetic wounds, a biocompatible gelatin methacryloyl (GelMA) hydrogel was employed to load and sustainably release AS at the wound site. Interestingly, AS‐loaded GelMA hydrogel (Gel‐AS) reduced M1 macrophage infiltration and downregulated the expression of ferritin heavy chain 1 (FTH1) and FPN1 proteins, facilitating diabetic wound healing process. Our results provide insight into a new therapeutic intervention to treat excessive inflammation‐related diseases.

## 2 Results

### 2.1 AS Reduced the Number of M1 Macrophages and Inhibited the Secretion of Proinflammatory Cytokines

**FIGURE 2:**
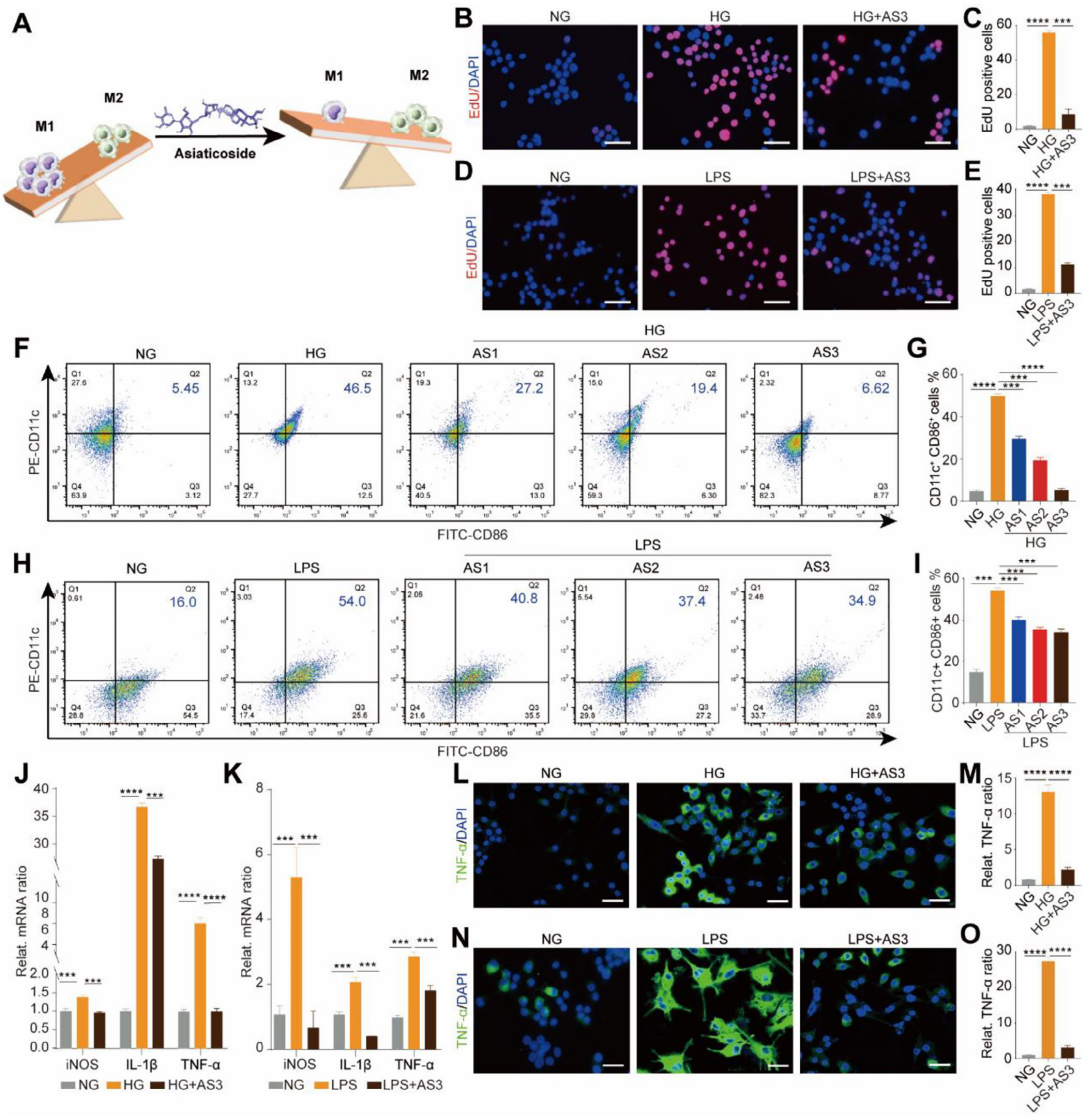
AS reduced the number of M1 macrophages and inhibited the secretion of proinflammatory cytokines. (A) Schematic representing the immunomodulation potential of AS in macrophages. (B) Fluorescent images and (C) the number of EdU‐positive cells (red fluorescence) demonstrating the proliferation behavior of NG macrophages, HG‐M1 macrophages, and AS‐treated HG‐M1 macrophages (Mean ± SD; one‐way ANOVA, *n* = 3). Scale bar, 100 µm. (D) Fluorescent images and (E) the number of EdU positive cells (red fluorescence) demonstrating the proliferation behavior of NG macrophages, LPS‐M1 macrophages, and AS‐treated LPS‐M1 macrophages (mean ± SD; one‐way ANOVA, *n* = 3). Scale bar, 100 µm. (F) FCM images and (G) quantitative analysis showing the proportion of CD11c^+^CD86^+^ cells in NG macrophages, HG‐M1 macrophages, and AS‐treated HG‐M1 macrophages (mean ± SD; one‐way ANOVA, *n* = 3). (H) FCM images and (I) quantitative analysis showing the proportion of CD11c^+^CD86^+^ cells in NG macrophages, LPS‐M1 macrophages, and AS‐treated LPS‐M1 macrophages (mean ± SD; one‐way ANOVA, *n* = 3). (J,K) qRT‐PCR results displaying the expression levels of iNOS, IL‐1β, and TNF‐α genes in macrophages with different treatments (mean ± SD; one‐way ANOVA, *n* = 3). (L) Fluorescent images and (M) quantitative statistics reflecting the expression of TNF‐α protein in NG macrophages, HG‐M1 macrophages, and AS‐treated HG‐M1 macrophages (mean ± SD; one‐way ANOVA, *n* = 3). Scale bar, 100 µm. (N) Fluorescent images and (O) quantitative statistics demonstrating the expression of TNF‐α protein in NG macrophages, LPS‐M1 macrophages, and AS‐treated LPS‐M1 macrophages (mean ± SD; one‐way ANOVA, *n* = 3). Scale bar, 100 µm. The cell nucleus was stained with DAPI (blue fluorescence). Statistically significant differences between groups are indicated as follows: ***p < 0.001, and ****p < 0.0001.

To obtain proinflammatory M1 macrophages (CD11c^+^CD86^+^), RAW264.7 cells (a type of murine macrophages) stimulated with HG (50 mM glucose) or LPS (100 ng mL^−1^), are called HG‐M1 (Figure S1A,B, Supporting Information) or LPS‐M1 (Figure S1C,D, Supporting Information) macrophages. Then, we investigated the effects of AS on the proliferation function of HG‐M1 or LPS‐M1 macrophages (Figure 2A). Three concentrations of AS including 0.001 mM (AS1), 0.01 mM (AS2), and 0.1 mM (AS3) were used to treat HG‐M1 macrophages. As shown in Figure S2A, AS reduced the viability of HG‐M1 macrophages in a dose‐dependent manner with AS3 exerting the most obvious inhibitory effect. As expected, AS3 also significantly reduced the viability of LPS‐M1 macrophages (Figure S2B, Supporting Information). The EdU assay showed that the number of EdU positive cells was reduced by AS3 treatment compared with that in the HG‐M1 macrophages (Figure 2B,C) and LPS‐M1 macrophages (Figure 2D,E). Next, the flow cytometry (FCM) assay was performed to determine the proportion of CD11c^+^CD86^+^ cells treated with AS1, AS2, and AS3. The results indicated that AS reduced the proportion of CD11c^+^CD86^+^ cells among HG‐M1 (Figure 2F,G) and LPS‐M1 (Figure 2H,I) macrophages in a dose‐dependent manner. AS3 was the most effective concentration to reduce the number of M1 macrophages. Meanwhile, the proportion of CD163^+^ cells in AS‐treated LPS‐M1 macrophages remains unchanged compared with untreated LPS‐M1 macrophages (Figure S3, Supporting Information). Additionally, we analyzed the effect of AS on the cell viability and the proportion of CD206^+^CD163^+^ cells in interleukin‐4 (IL‐4)‐treated macrophages (M2 macrophages). As shown in Figure S4A, AS did not reduce the cell viability of M2 macrophages. Furthermore, AS also showed negligible influence on the proportion of CD206^+^CD163^+^ cells (Figure S4B,C, Supporting Information). These results suggest that AS can specifically reduce the number of M1 macrophages, while shows negligible effect on the number of M2 macrophages.

Subsequently, we investigated whether AS could inhibit the secretion of proinflammatory cytokines from HG‐M1 and LPS‐M1 macrophages. As shown in Figure 2J,K, the mRNA levels of proinflammatory genes including tumor necrosis factor‐α (TNF‐α), inducible nitric oxide synthase (iNOS), and interleukin‐1β (IL‐1β) were significantly increased in HG‐M1 and LPS‐M1 macrophages compared with those in macrophages without HG and LPS induction. However, AS3 significantly downregulated the mRNA levels of TNF‐α, iNOS, and IL‐1β genes. Additionally, the immunofluorescence staining was used to evaluate the protein expression of TNF‐α, iNOS, and IL‐1β in AS3‐treated HG‐M1 and LPS‐M1 macrophages. The results showed that the increased expression TNF‐α, iNOS, and IL‐1β proteins in HG‐M1 (Figure 2L,M; Figure S5A,B,E,F, Supporting Information) and LPS‐M1 (Figure 2N,O; Figure S5C,D,G,H, Supporting Information) macrophages was downregulated heavily with the introduction of AS3. Collectively, these results demonstrated that AS reduced the number of M1 macrophages induced by HG and LPS, and inhibited the secretion of proinflammatory cytokines.

### 2.2 Ferroptosis was Responsible for AS‐Induced Functional Downregulation of HG‐M1 Macrophages

Based on the above results, we speculate that ferroptosis may be involved in a decrease in the number of M1 macrophages. Therefore, we pretreated HG‐M1 macrophages with deferoxamine (DFO, a ferroptosis inhibitor) before treating them with AS and assessed their cell viability. Interestingly, pretreating HG‐M1 macrophages with 8 µM DFO for 6 h significantly antagonized AS3‐reduced cell viability of HG‐M1 macrophages, suggesting that ferroptosis was involved in the regulatory function of AS on HG‐M1 macrophages (Figure S6A, Supporting Information). Additionally, to exclude the role of other types of programmed cell death, the apoptosis inhibitor (carbobenzoxy‐valyl‐alanyl‐aspartyl‐[O‐methyl]‐fluoromethylketone, ZVD) and the necrosis inhibitor (necrostatin‐1, NEC‐1) were also used to pretreat HG‐M1 macrophages. The results showed the pretreatment of HG‐M1 macrophages with ZVD or NEC‐1 displayed negligible effect on AS3‐reduced cell viability (Figure S6B,C, Supporting Information).

**FIGURE 3:**
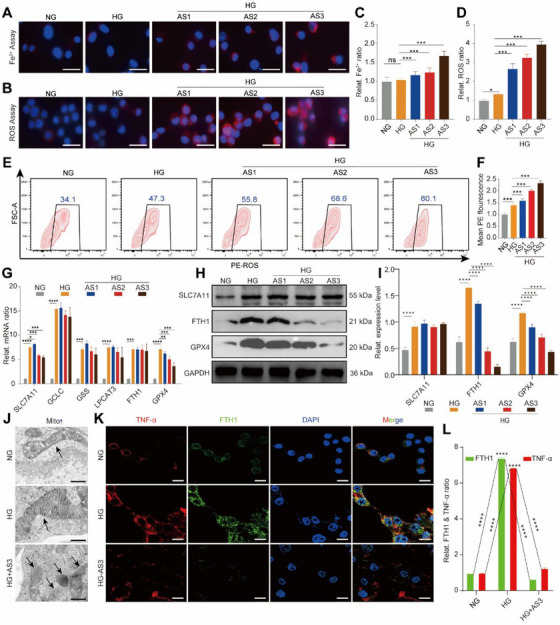
Ferroptosis was responsible for AS‐induced functional downregulation of HG‐M1 macrophages. (A) Representing fluorescent images showing Fe^2+^ concentration in NG macrophages, HG‐M1 macrophages, and AS‐treated HG‐M1 macrophages. Scale bar, 10 µm. (B) Fluorescent images showing ROS levels in macrophages with the above treatments. Scale bar, 10 µm. (C) Quantitative analysis of Fe^2+^ concentration in (A) (mean ± SD; one‐way ANOVA, *n* = 3). (D) Quantitative analysis of ROS level in (C) (mean ± SD; one‐way ANOVA, *n* = 3). (E) FCM images and (F) corresponding statistical analysis showing ROS levels in macrophages with the above treatments (mean ± SD; one‐way ANOVA, *n* = 3). (G) qRT‐PCR results displaying the expression levels of ferroptosis‐associated genes, including SLC7A11, GCLC, GSS, LPCAT3, FTH1, and GPX4, in macrophages with the above treatments (mean ± SD; one‐way ANOVA, *n* = 3). (H) Western blotting images and (I) quantitative band intensities showing the expression levels of SLC7A11, FTH1 and GPX4 proteins in macrophages with the above treatments (mean ± SD; one‐way ANOVA, *n* = 3). (J) TEM images showing the morphology deformation of mitochondria (black arrows) in macrophages with the above treatments (mean ± SD; one‐way ANOVA, *n* = 3). Scale bar, 500 nm. (K) Immunofluorescence co‐staining images and (L) corresponding relative fluorescent intensity analysis showing the relative ratio of FTH1 (green fluorescence) and TNF‐α (red fluorescence) in macrophages with above treatments (mean ± SD; one‐way ANOVA, *n* = 3). Scale bar, 10 µm. The cell nucleus was dyed with DAPI (blue fluorescence). Statistically significant differences between groups are indicated as follows: ns, not significant, * p < 0.05, ***p < 0.001, and ****p < 0.0001.

To further confirm the AS‐induced ferroptosis of HG‐M1 macrophages, we observed the intracellular free Fe^2+^, ROS and glutathione (GSH) levels, the expression of ferroptosis‐related proteins, the morphology of mitochondria, and the co‐localization of TNF‐α and FTH1, systematically. First, the obvious accumulations of free Fe^2+^ (Figure 3A,C) and ROS (Figure 3B,D–F) were observed in AS‐treated HG‐M1 macrophages. Concurrently, a notable decline in GSH levels was observed in AS‐treated HG‐M1 macrophages (Figure S7, Supporting Information). Next, the expression of ferroptosis‐related proteins [34] including solute carrier family 7 member 11 (SLC7A11), glutamate‐cysteine ligase catalytic subunit (GCLC), glutathione synthetase (GSS), lysophosphatidylcholine acyltransferase 3 (LPCAT3), FTH1, and glutathione peroxidase 4 (GPX4) were detected in our experiments. The qRT‐PCR results indicated that the mRNA levels of GPX4 and SLC7A11 in HG‐M1 macrophages were downregulated by adding AS, while those of GSS, GCLC, LPCAT3, and FTH1 showed a weak change (Figure 3G). Interestingly, the protein expression level of SLC7A11 was not reduced obviously by AS, while that of FTH1 and GPX4 was downregulated heavily (Figure 3H,I). Furthermore, we detected the effect of AS on the enzymatic catalytic activities of GSS, GPX4, and LPCAT3 in HG‐M1 macrophages. As shown in Figure S8, the enzymatic catalytic activities of GSS, GPX4, and LPCAT3 were enhanced by 3–4 times in HG conditions. However, AS showed disparate effects on the enzymatic catalytic activities of GSS, GPX4, and LPCAT3. Specifically, AS only resulted in a dose‐dependent reduction in the enzymatic catalytic activity of GPX4, while showed limited effect on the activities of GSS and LPCAT3 in HG‐M1 macrophages. Another feature of ferroptosis is the deformation of mitochondria within the cells. As shown in transmission electron microscopy (TEM) images in Figure 3J, the mitochondria were significantly shrunken and the mitochondrial cristae almost disappeared (shown by black arrows) in AS3‐treated HG‐M1 macrophages. Moreover, the co‐staining of TNF‐α (red fluorescence) and FTH1 (green fluorescence) showed that the protein expressions of TNF‐α and FTH1 in HG‐M1 macrophages were inhibited synchronously by AS3 (Figure 3K,L).

Subsequently, the inhibitor (DFO) and the activator (Erastin) of ferroptosis were used to illustrate the relationship between ferroptosis and the function of HG‐M1 macrophages induced by AS. The results demonstrated that the AS3‐induced ferroptosis of HG‐M1 macrophages was antagonized by pretreatment with DFO, as shown by partially restored GPX4 protein expression (Figure S9A,B, Supporting Information). Simultaneously, AS3‐reduced cell viability was improved by the pretreatment with DFO (Figure S9C, Supporting Information). Furthermore, the pretreatment of AS3‐induced HG‐M1 macrophages with Erastin obviously decreased the expression of GPX4, demonstrating the aggravation of AS3‐induced ferroptosis (Figure S9D,E, Supporting Information). As expected, pretreatment with Erastin further reduced the viability of AS3‐induced HG‐M1 macrophages (Figure S9F, Supporting Information). Furthermore, the effects of DFO and Erastin on the expression of proinflammatory cytokines in AS3‐induced HG‐M1 macrophages were also observed. As shown in Figure S9G, the mRNA levels of TNF‐α, iNOS, and IL‐1β were partially restored by the pretreatment with DFO and downregulated by the pretreatment with Erastin. Additionally, the protein expression of TNF‐α in AS3‐induced HG‐M1 macrophages treated with DFO or Erastin showed a similar trend to the mRNA results (Figure S9H,I, Supporting Information).

Overall, AS activated ferroptosis in HG‐M1 macrophages and inhibited their functions. The ferroptosis inhibitor could reverse the inhibitory effect of AS on the function of HG‐M1 macrophages. The ferroptosis activator exacerbated AS3‐induced ferroptosis and downregulated the function of AS‐induced HG‐M1 macrophages. In particular, M1 macrophages have been reported to exhibit resistance to RSL3‐induced ferroptosis compared to M0 and M2 macrophages [35]. To further investigate this paradox, we subsequently explored the possible molecular mechanisms by which AS induced ferroptosis specifically in HG‐M1/LPS‐M1 macrophages and not in IL‐4‐M2 macrophages.

### 2.3 Ferritinophagy‐Dependent Degradation of FTH1 was Involved in AS‐Induced Ferroptosis of HG‐M1 Macrophages

**FIGURE 4:**
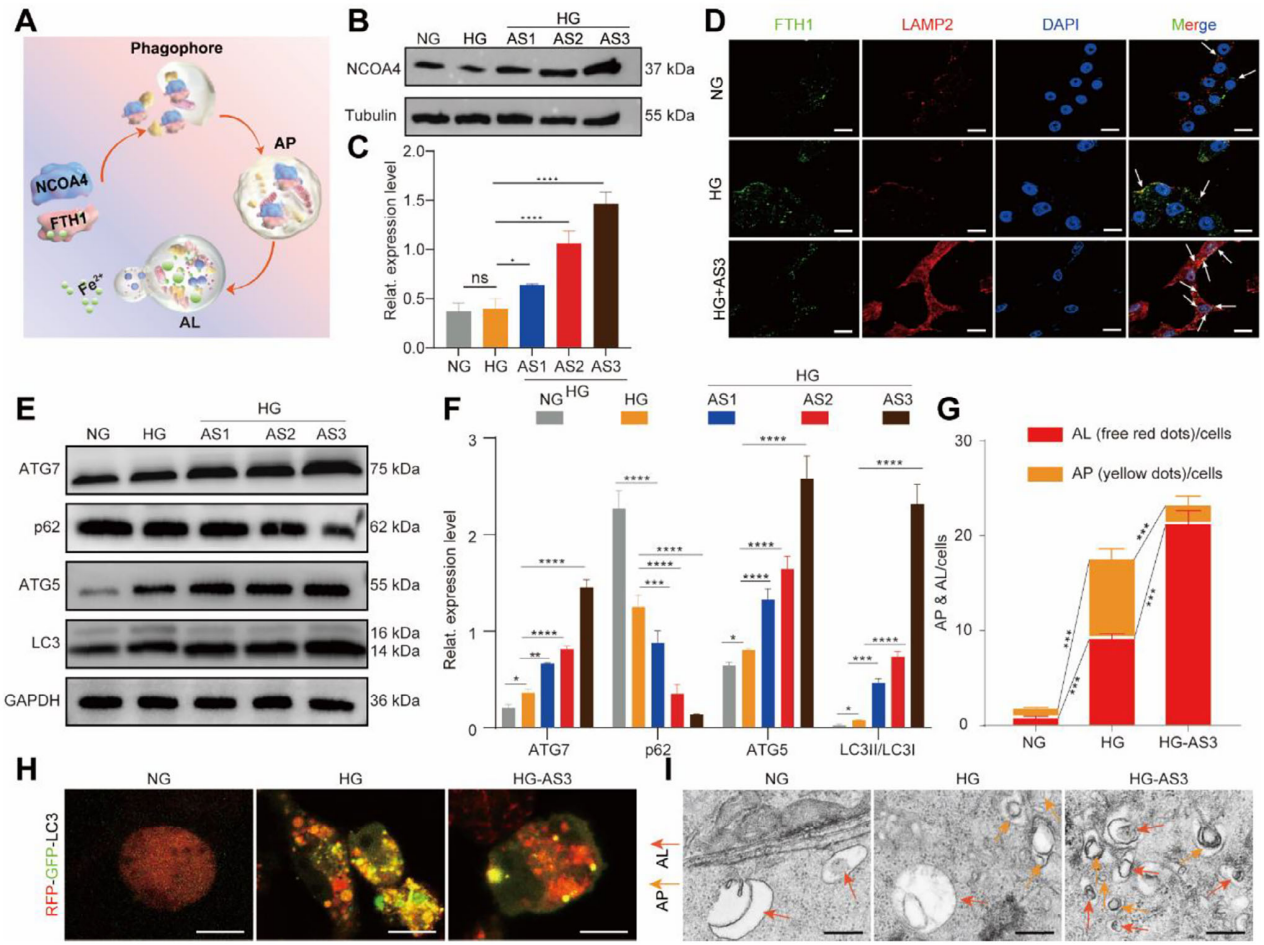
Ferritinophagy‐dependent degradation of FTH1 was involved in AS‐induced ferroptosis of HG‐M1 macrophages. (A) Schematic representing the degradation process of FTH1, which was bound to NCOA4 and transported to ALs by APs. (B) Western blotting images and (C) quantitative band intensities showing the expression level of NCOA4 protein in NG macrophages, HG‐M1 macrophages, and AS‐treated HG‐M1 macrophages (mean ± SD; one‐way ANOVA, *n* = 3). (D) Fluorescent images of co‐staining (indicated by white arrows) of FTH1 (green fluorescence) and LAMP2 (red fluorescence) showing the activation of ferritinophagy in macrophages with above treatments (mean ± SD; one‐way ANOVA, *n* = 5). Scale bar, 10 µm. (E) Western blotting images and (F) quantitative band intensities showing the expression levels of the ATG7, ATG5, p62, and LC3 proteins in macrophages with the above treatments (mean ± SD; one‐way ANOVA, *n* = 3). (G) Quantitative analysis of (H) showing the mean numbers of APs (indicated by yellow fluorescent dots) and ALs (indicated by red fluorescent dots) in the merged area per macrophage with the above treatments. (H) Ad‐mRFP‐GFP‐LC3 double fluorescence indicator showing the activation of autophagy in macrophages with the above treatments (mean ± SD; one‐way ANOVA, *n* = 3). Scale bar, 10 µm. (I) TEM images showing the number of APs (indicated by red arrows) and ALs (indicated by yellow arrows) in macrophages with above treatments. Scale bar, 500 nm. The cell nucleus was dyed with DAPI (blue fluorescence). Statistically significant differences between groups are indicated as follows: ns, not significant, * p < 0.05, **p < 0.01, ***p < 0.001, and ****p < 0.0001.

The above results showed that the mRNA level of FTH1 was not affected but the protein level was significantly downregulated in AS‐induced HG‐M1 macrophages, suggesting AS might lead to the degradation of FTH1 protein in HG‐M1 macrophages. Reportedly, ferritinophagy is the major degradation pathway of FTH1 protein, which requires the participation of nuclear receptor coactivator 4 (NCOA4). The conjugated FTH1‐NCOA4 is encapsulated into autophagosomes (APs) and delivered to autolysosomes (ALs) for degradation (Figure 4A). Degradation of FTH1 protein leads to the release of stored Fe^2+^, leading to ferritinophagy‐mediated ferroptosis [36]. To determine whether ferritinophagy is involved in FTH1 protein degradation in AS‐induced HG‐M1 macrophages, we examined the expression of NCOA4, the activation of ALs, and the expression of autophagy‐related proteins. First, we observed that the expression of the NCOA4 protein inside HG‐M1 macrophages was upregulated by AS in a dose‐dependent manner (Figure 4B,C). Specifically, AS3 demonstrated the most significant enhancement on NCOA4, surpassing the AS1 and AS2 groups. Next, to illustrate the degradation of FTH1 by the autophagy‐dependent pathway, the expression of FTH1 (green fluorescence) was co‐localized with the lysosomal‐associated membrane protein 2 (LAMP2, the biomarker of ALs). As shown in Figure 4D, FTH1 protein was delivered to ALs (red fluorescence) and degraded in AS‐treated HG‐M1 macrophage. Then, the expression levels of the autophagy‑related gene‐5 (ATG5), ATG7, light chain 3 (LC3II)/LC3I (positive regulatory correlation), and p62 (negative regulatory correlation) were detected. The results showed that AS upregulated the expression of ATG5, ATG7, and LC3II/LC3I and downregulated the expression of p62 at both protein (Figure 4E,F) and mRNA levels (Figure S10, Supporting Information). Additionally, tandem fluorescent mRFP‐GFP‐LC3 assays showed that more APs entered ALs to be degraded in AS3‐induced HG‐M1 macrophages (Figure 4G,H). Furthermore, the formation and distribution of APs and ALs were observed by TEM (Figure 4I). The number of ALs (represented by red arrows) was increased and the number of APs was decreased, indicating high autophagic flux in AS‐induced HG‐M1 macrophages.

**FIGURE 5:**
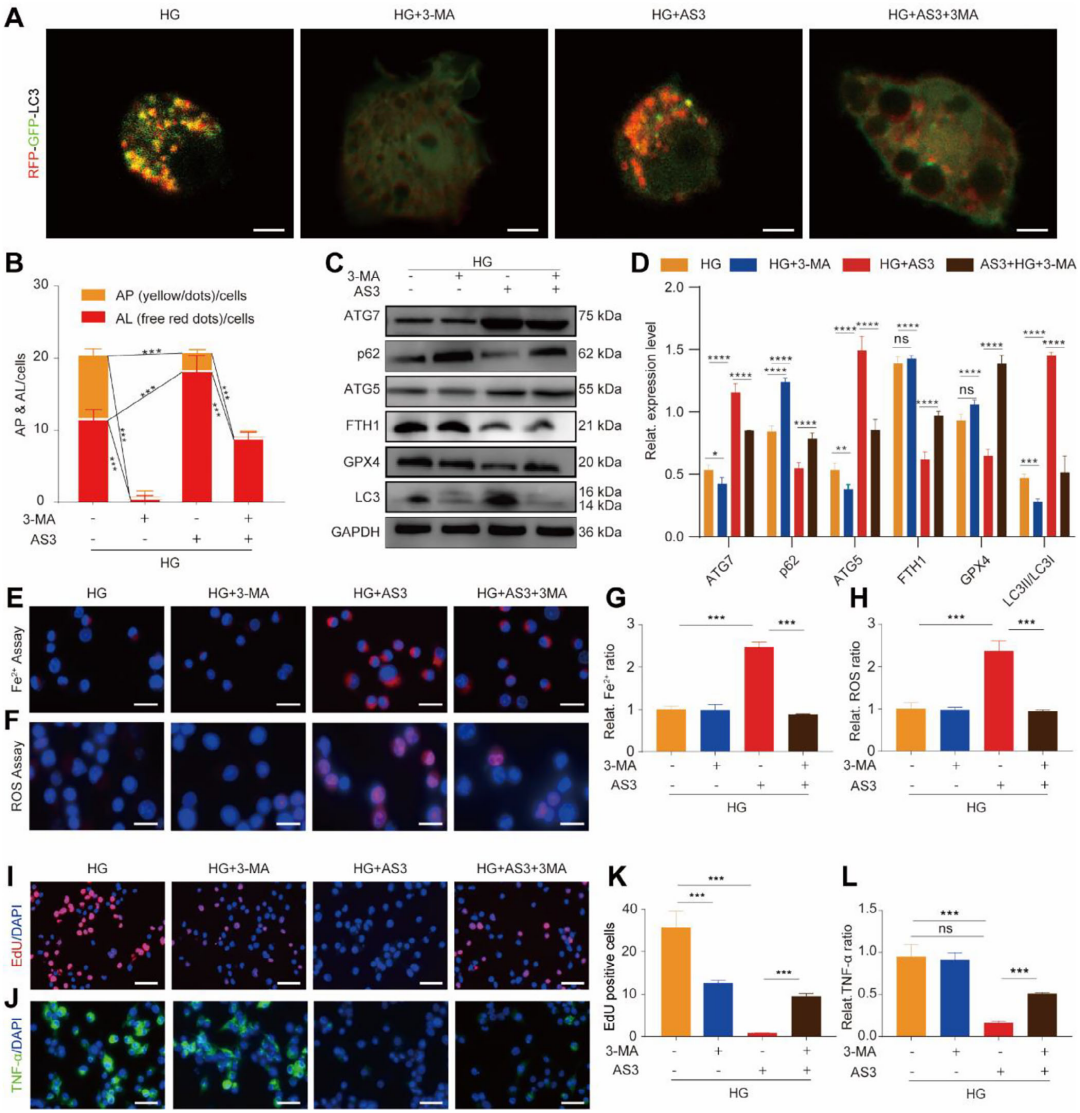
3‐MA antagonized AS‐activated ferritinophagy, alleviating ferroptosis of HG‐M1 macrophages. (A) Ad‐mRFP‐GFP‐LC3 double fluorescence indicator and (B) corresponding quantitative analysis showing the effect of 3‐MA‐pretreatment on autophagy flux within HG‐M1 macrophages with or without post‐treatment of AS3 (mean ± SD; one‐way ANOVA, *n* = 5). Scale bar, 10 µm. (C) Western blotting images and (D) quantitative band intensities indicating the expression levels of autophagy‐associated proteins (ATG7, p62, ATG5, and LC‐3) and ferroptosis‐associated proteins (FTH1 and GPX4) in HG‐M1 macrophages with the above treatments (mean ± SD; one‐way ANOVA, *n* = 3). (E) Representing fluorescent images showing Fe^2+^ concentration inside HG‐M1 macrophages with the above treatments. Scale bar, 5 µm. (F) Representing fluorescent images of ROS level inside HG‐M1 macrophages with above treatments. Scale bar, 5 µm. (G) Quantitative analysis of Fe^2+^ concentration in (E) (mean ± SD; one‐way ANOVA, *n* = 3). (H) Quantitative analysis of ROS level in (F) (mean ± SD; one‐way ANOVA, *n* = 3). (I) Fluorescent images of EdU assay (red fluorescence) showing the proliferation behavior of macrophages with the above treatments. Scale bar, 100 µm. (J) Fluorescent images reflecting TNF‐α expression levels in macrophages with above treatments. The cell nucleus was dyed with DAPI (blue fluorescence). Scale bar, 100 µm. (K) Quantitative result of EdU assay in (I) (mean ± SD; one‐way ANOVA, *n* = 3). (L) Quantitative analysis reflecting TNF‐α expression levels in (J) (mean ± SD; one‐way ANOVA, *n* = 3). Statistically significant differences between groups are indicated as follows: *p < 0.05, **p < 0.01, ***p < 0.001, and ****p < 0.0001.

Next, the autophagy inhibitor 3‐methyladenine (3‐MA) was used to block autophagy flux in AS‐induced HG‐M1 macrophages (Figure 5A,B). We observed that the autophagy‐related positive regulatory proteins including ATG5, ATG7, and LC3II/LC3I were downregulated and the autophagy‐related negative regulatory protein p62 was upregulated. As expected, after blocking autophagy flux with 3‐MA, the expression of FTH1 and GPX4 was restored (Figure 5C,D) and the levels of free Fe^2+^ (Figure 5E,G) and ROS (Figure 5F,H) were significantly reduced compared with those in HG‐M1 macrophages treated with AS3 alone. Then, we observed the effects of blocking autophagy and ferroptosis by 3‐MA on the proliferation and secretion capacities of AS‐induced HG‐M1 macrophages. Apparently, pretreatment of 3‐MA restored cell viability of HG‐M1 macrophages reduced by AS3 (Figure S11, Supporting Information). The number of EdU‐positive cells reduced by AS3 was partially suppressed with pretreatment with 3‐MA (Figure 5I,K). Simultaneously, the secretion of TNF‐α, iNOS, and IL‐1β was also partially restored by 3‐MA in AS‐induced HG‐M1 macrophages (Figure 5J,L and Figure S12, Supporting Information).

Subsequently, the autophagy activator rapamycin (RAPA) was also used to activate autophagy in AS‐induced HG‐M1 macrophages (Figure S13A,B, Supporting Information). We observed that the protein expression levels of ATG5, ATG7, and LC3II/LC3I was upregulated and that of p62 was downregulated. As expected, the ferroptosis‐related proteins such as FTH1 and GPX4 were decreased after RAPA activated autophagy (Figure S13C,D, Supporting Information). Accordingly, the levels of free Fe^2+^ (Figure S13E,G, Supporting Information) and ROS (Figure S13F,H, Supporting Information) were increased by RAPA in AS‐induced HG‐M1 macrophages. Then, we observed the effects of RAPA on the proliferation and secretion capacities of AS‐induced HG‐M1 macrophages. The results showed that RAPA enhanced the inhibitory function of AS on the proliferation behaviors (Figures S14 and S15A,C, Supporting Information) and the secretion of proinflammatory cytokines of HG‐M1 macrophages (Figure S15B,D,E, Supporting Information).

Collectively, these results demonstrated that AS activated ferritinophagy and led to the degradation of FTH1 in HG‐M1 macrophages. The autophagy inhibitor 3‐MA blocked AS‐induced ferroptosis and increased the proliferation and secretion capacities of AS‐induced HG‐M1 macrophages. The autophagy activator RAPA had the opposite effect on ferroptosis and the function of AS‐induced HG‐M1 macrophages.

### 2.4 Downregulation of FPN1 Protein was Also Involved in AS‐Induced Ferroptosis of HG‐M1 Macrophages

**FIGURE 6:**
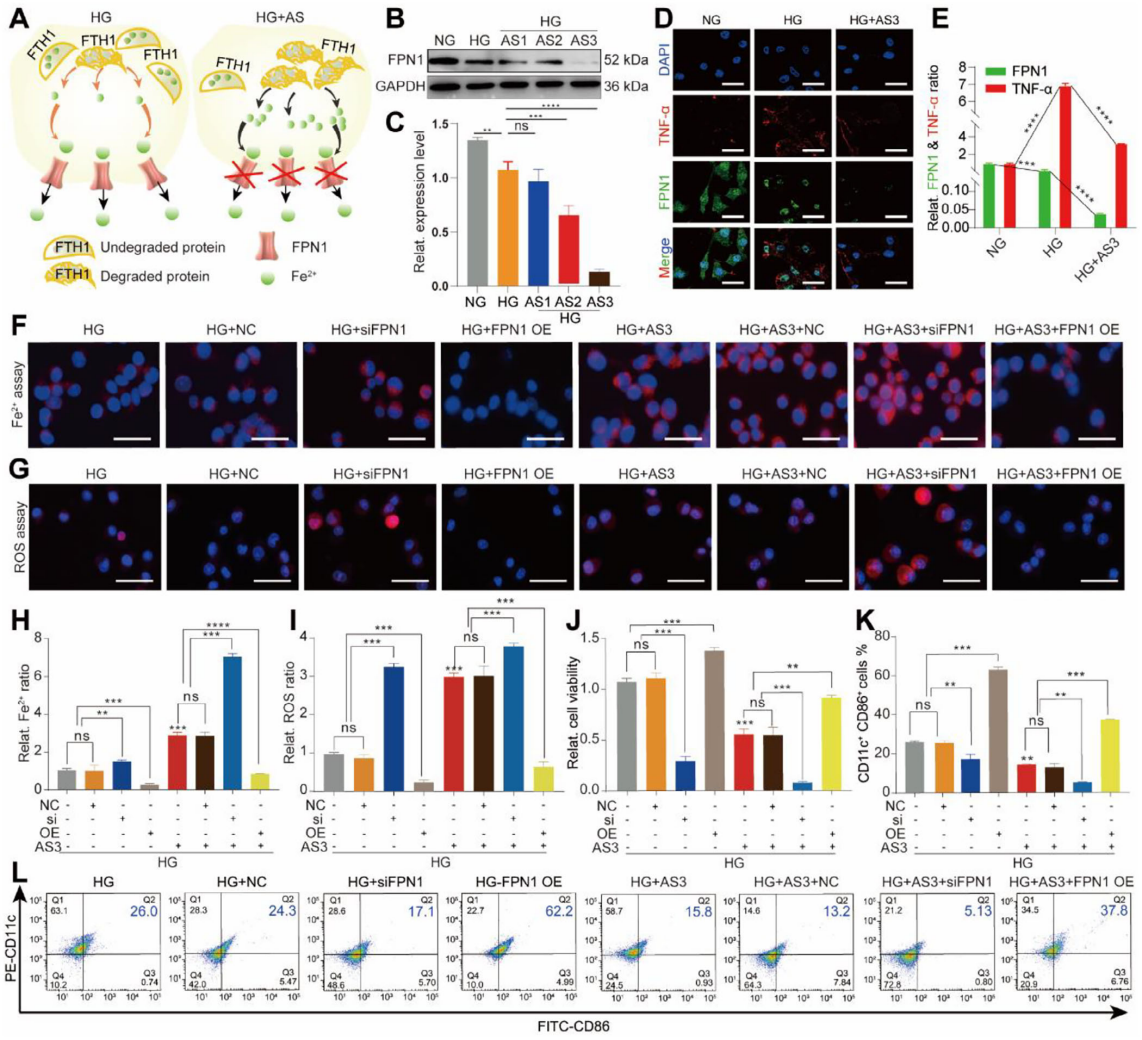
Downregulation of FPN1 protein was also involved in AS‐induced ferroptosis of HG‐M1 macrophages. (A) Schematic representation showing that AS blocked the transportation of free Fe^2+^ to out of HG‐M1 macrophages by downregulating the expression of FPN1 protein. (B) Western blotting images and (C) quantitative band intensities showing the expression level of FPN1 protein in NG macrophages, HG‐M1 macrophages, and AS‐treated HG‐M1 macrophages (mean ± SD; one‐way ANOVA, *n* = 3). (D) Immunofluorescence co‐staining of TNF‐α and FPN1 proteins and (E) corresponding analysis of relative fluorescent intensity in macrophages with the above treatments (mean ± SD; one‐way ANOVA, *n* = 3). Scale bar, 20 µm. (F) Representative fluorescent images of Fe^2+^ accumulation in HG‐M1 macrophages with different groups. Scale bar, 50 µm. (G) Representative fluorescent images of ROS accumulation in HG‐M1 macrophages exposed to different groups. Scale bar, 20 µm. (H) Corresponding quantitative analysis of Fe^2+^ accumulation in (F). (I) Corresponding quantitative analysis of ROS accumulation in (G) (mean ± SD; one‐way ANOVA, *n* = 3). (J) Relative cell viability of HG‐M1 macrophages exposed to different groups (mean ± SD; one‐way ANOVA, *n* = 6). (K) Quantitative analysis and (L) FCM results showing the proportion of CD11c^+^CD86^+^ cells in HG‐M1 macrophages exposed to different groups (mean ± SD; one‐way ANOVA, *n* = 3). The cell nucleus was dyed with DAPI (blue fluorescence). Statistically significant differences between groups are indicated as follows: ns, not significant, **p < 0.01, ***p < 0.001, and ****p < 0.0001.

FPN1, as a specific iron transfer protein, is reported to account for pumping out excessive free Fe^2+^ from cells [37]. Hence, blocking the transportation of free Fe^2+^ could be another mechanism for AS to activate ferroptosis of HG‐M1 macrophages (Figure 6A). To prove our hypothesis, we first detected the expression of FPN1 and found that FPN1 expression were reduced markedly by AS in a dose‐dependent manner (Figure S16, Supporting Information and Figure 6B,C). The co‐staining of TNF‐α (red fluorescence) and FPN1 (green fluorescence) showed the correlation between FPN1 and M1 macrophages (Figure 6D,E).

To further understand the crucial role of FPN1 protein in determining AS‐induced ferroptosis in HG‐M1 macrophages, we overexpressed or silenced the FPN1 gene in AS‐induced HG‐M1 macrophages transfected with FPN1 plasmids (Figure S17A–C, Supporting Information) and FPN1 siRNAs (Figure S17D–F, Supporting Information). Then, the influence of overexpressing or silencing FPN1 protein on AS‐mediated ferroptosis in HG‐M1 macrophages was observed. As shown in Figure 6F,H, the concentration of free Fe^2+^ in AS‐induced HG‐M1 macrophages was increased by siFPN1 and decreased by FPN1 plasmids. The change of ROS concentration was similar to that of free Fe^2+^ (Figure 6G,I).

Next, we investigated the effects of overexpressing or silencing the FPN1 protein on AS‐regulated proliferation behavior, the proportion of CD11c^+^CD86^+^ cells, and the production of proinflammatory cytokines. As shown in Figure S18, the number of EdU positive cells among AS‐induced HG‐M1 macrophages was decreased by FPN1 siRNAs and increased by FPN1 plasmids. The similar tendency was observed in the cell viability of AS‐induced HG‐M1 macrophages (Figure 6J). In addition, suppression of FPN1 protein expression significantly downregulated the expressions of iNOS, IL‐1β, and TNF‐α, and overexpressing the FPN1 protein increased the expression of the above‐mentioned proinflammatory cytokines in AS‐induced HG‐M1 macrophages (Figure S19, Supporting Information). Furthermore, the ability of AS to reduce the proportion of CD11c^+^CD86^+^ could be further enhanced by siFPN1 and neutralized by overexpressing FPN1 protein (Figure 6K,L).

Taken together, AS downregulated of FPN1 protein in HG‐M1 macrophages, which probably was another mechanism for AS‐induced ferroptosis of HG‐M1 macrophages.

### 2.5 AS‐Loaded GelMA Hydrogel Facilitated Diabetic Wound Healing

**FIGURE 7:**
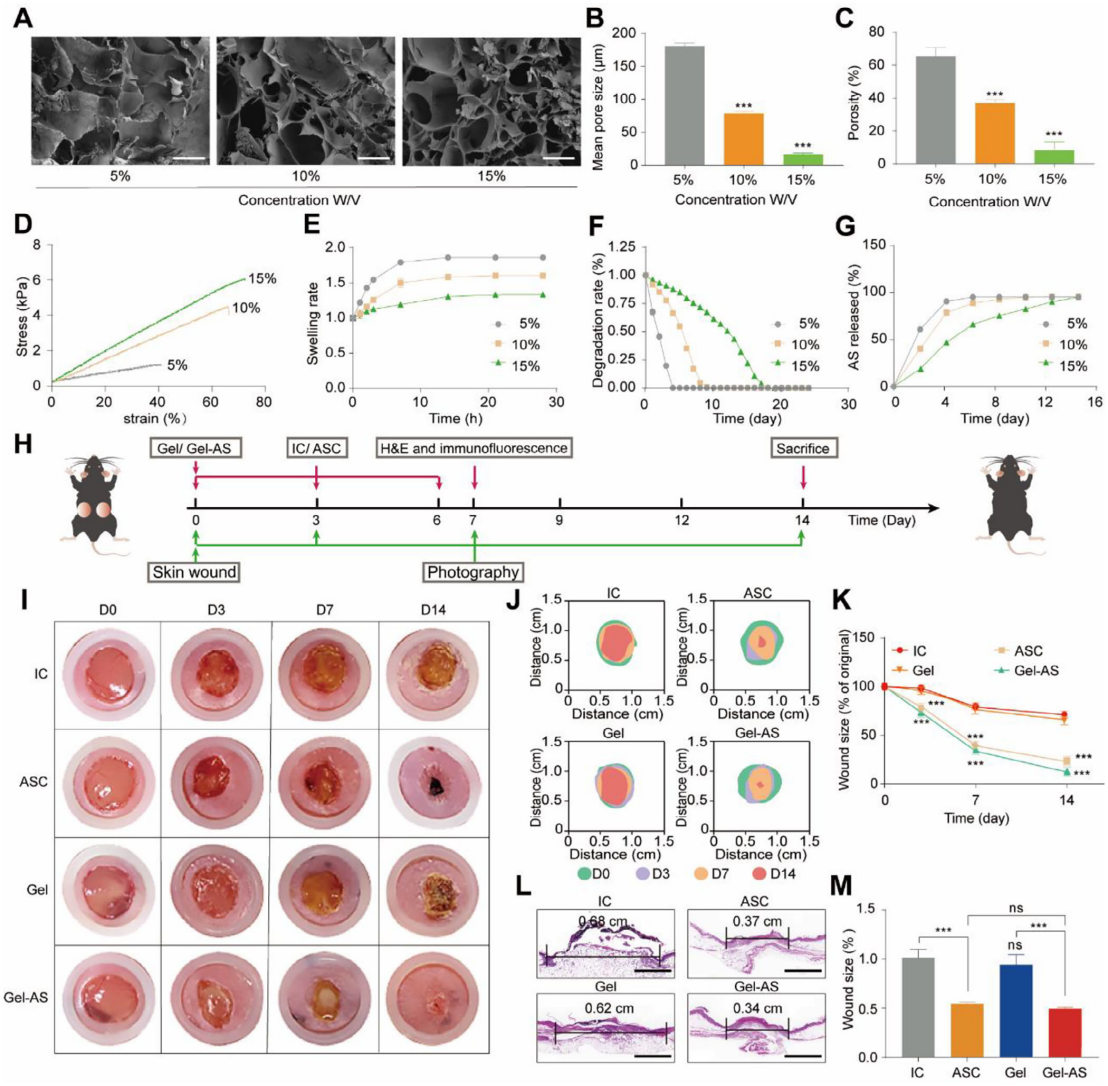
AS‐loaded GelMA hydrogel facilitated diabetic wound healing. (A) SEM images, (B) pore size, (C) porosity, (D) stress–strain curves, (E) swelling rate, and (F) degradation rate of 5%‐Gel, 10%‐Gel, and 15%‐Gel (mean ± SD; one‐way ANOVA, *n* = 3). Scale bar, 200 µm for (A). (G) Release behavior of AS from 5%‐Gel, 10%‐Gel, and 15%‐Gel (mean ± SD; one‐way ANOVA, *n* = 3). (H) The wounding and treatment schematics on the back of diabetic mice. (I) Optical images, (J) wound traces, and (K) quantitative results of the wounds treated with IC, ASC, Gel and Gel‐AS (mean ± SD; one‐way ANOVA, *n* = 6). (L) H&E images and (M) corresponding quantitative analysis of wounds from the above groups on D7 (mean ± SD; one‐way ANOVA, *n* = 6). Scale bar, 2 mm. Statistically significant differences between groups are indicated as follows: ns, not significant, ***p < 0.001.

The ability of AS to activate ferroptosis and reduce the number of HG‐M1 macrophages inspired us to explore the therapeutic potential of AS‐based wound dressings. In our study, we encapsulated AS into GelMA hydrogels and optimized the release time of AS by regulating the monomer concentrations of GelMA (5%‐Gel, 10%‐Gel, and 15%‐Gel). When the concentration of Gel increased from 5% to 15%, the pore size of Gel was decreased by about 90%, as shown in the scanning electron microscope (SEM) images (Figure 7A,B) and the porosity was decreased by about 55.3% (Figure 7C). Meanwhile, the mechanical properties of Gel including the maximum tensile strain and the maximum tensile strength were increased to 73.7% and 6 kPa, when the concentration was 15% (Figure 7D). Furthermore, a higher degree of crosslinking achieved by higher monomer concentrations also resulted in lower swelling and degradation rate because of the restricted diffusion of water molecules. Specifically, the swelling rates of 5%‐Gel, 10%‐Gel, and 15%‐Gel were increased by about 0.73‐fold, 0.52‐fold, and 0.27‐fold at 24 h compared with their initial swelling rates (Figure 7E). 5%‐Gel, 10%‐Gel, and 15%‐Gel were completely degraded in buffer with collagenase on D4, D8, and D17, respectively (Figure 7F). More importantly, the incorporation of AS did not significantly change the chemical structure and elemental composition of the Gel hydrogel (Figure S20, Supporting Information). Another effect of a high crosslinking degree is to prolong the sustained release time of AS. Standard curves were generated by high‐performance liquid chromatography (HPLC in Figure S21, Supporting Information). AS was completely released from 15%‐Gel on D16, while the time for 10%‐Gel and 5%‐Gel was D8 and D5, respectively (Figure 7G). Considering that AS exerts its anti‐inflammatory effects during the inflammatory phase, the release time of D8 is sufficient, therefore 10%‐Gel was elected as the carrier of AS (designated as Gel‐AS) in forthcoming experiments.

Meanwhile, we also evaluated the in vitro and in vivo biocompatibility of Gel‐AS according to previous reports [38]. As shown in Figure S22A,B, we observed that the immersion solution of Gel (Gel‐IS) and Gel‐AS (Gel‐AS‐IS) showed negligible effect on the cell viability of HUVECs and HDFs in HG conditions (HG‐HUVECs and HG‐HDFs). Furthermore, treating diabetic wounds with IC, ASC, Gel, and Gel‐AS did not result in obvious pathological damages to the major organs (heart, liver, spleen, lung, and kidney) of the mice (Figure S22C, Supporting Information). Therefore, Gel‐AS exhibited good biocompatibility both in vitro and in vivo. Epidemiological statistics indicate that *Staphylococcus aureus* is the predominant bacteria present in diabetic wounds [39]. Therefore, we tested the antibacterial effect of Gel‐AS‐IS against *S. aureus* using the colony counting method. The colony number of *S. aureus* was significantly decreased when it was incubated with Gel‐AS‐IS (Figure S23, Supporting Information). Reportedly, AS could dissolve the waxy membrane of bacteria to achieve an antibacterial effect [40]. Therefore, AS released from Gel‐AS showed clear antibacterial effects against *S. aureus*, preventing wound infection when applied to diabetic wounds for long periods of time.

To investigate the pro‐healing effect of Gel‐AS on diabetic wounds, full‐thickness wounds (the diameter of approximately 1 cm) were created on the backs of anaesthetized diabetic mice (db/db). Then, these mice were randomly divided into four groups: insert cream (IC), commercial asiaticoside cream (ASC), Gel, and Gel‐AS. The wounding and treatments of four groups were shown schematically in Figure 7H. Gel‐AS and Gel were applied only on D0, while IC and ASC were treated on D0, D3, and D6. The gross observation in Figure 7I–K indicated that the wounds treated with Gel‐AS showed the fastest healing rate compared with the other three groups. On D14, the unhealed area of Gel‐AS‐treated wounds was about 10%, while approximately 15% of the wound area remained unhealed for ASC group. In contrast, wounds treated with Gel and IC exhibited a greater extent of persistently unhealed wound area. Additionally, the wound sizes in hematoxylin and eosin (H&E)‐stained sections were measured in each group on D7 (Figure 7L,M). The size of unhealed wounds in both the IC and Gel groups was significantly larger than that in the ASC and Gel‐AS groups, being approximately twice as large. Therefore, Gel‐AS facilitated diabetic wound healing in vivo.

### 2.6 Gel‐AS Activated Ferroptosis of M1 Macrophages In Vivo

A long‐lasting inflammatory phase characterized by unbalanced M1/M2 ratio is recognized as the characteristic of chronic wounds [41]. Therefore, we investigated the anti‐inflammation role of Gel‐AS by evaluating the macrophage ratio of M1 and M2 phenotypes and examining the expression of TNF‐α at wound sites treated with different groups on D2 and D7. On D2, lots of F4/80^+^CD86^+^ cells (M1 macrophages) were observed at wound sites with all four treatments (Figure S24A, Supporting Information). No significant difference was observed in the number of F4/80^+^CD86^+^ macrophages (Figure S24D, Supporting Information). Furthermore, the expression of TNF‐α was relatively high at the wound sites with four treatments (Figure S24B,E, Supporting Information). Conversely, the number of F4/80^+^CD206^+^ cells (M2 macrophages) was almost invisible at wound sites with four treatments (Figure S24C,F, Supporting Information). Therefore, AS did not reduce the number of M1 macrophages or decrease the secretion of TNF‐α at wound sites on D2. All wounds exhibited typical inflammatory feature, characterized by enriched M1 macrophages and elevated TNF‐α expression.

**FIGURE 8:**
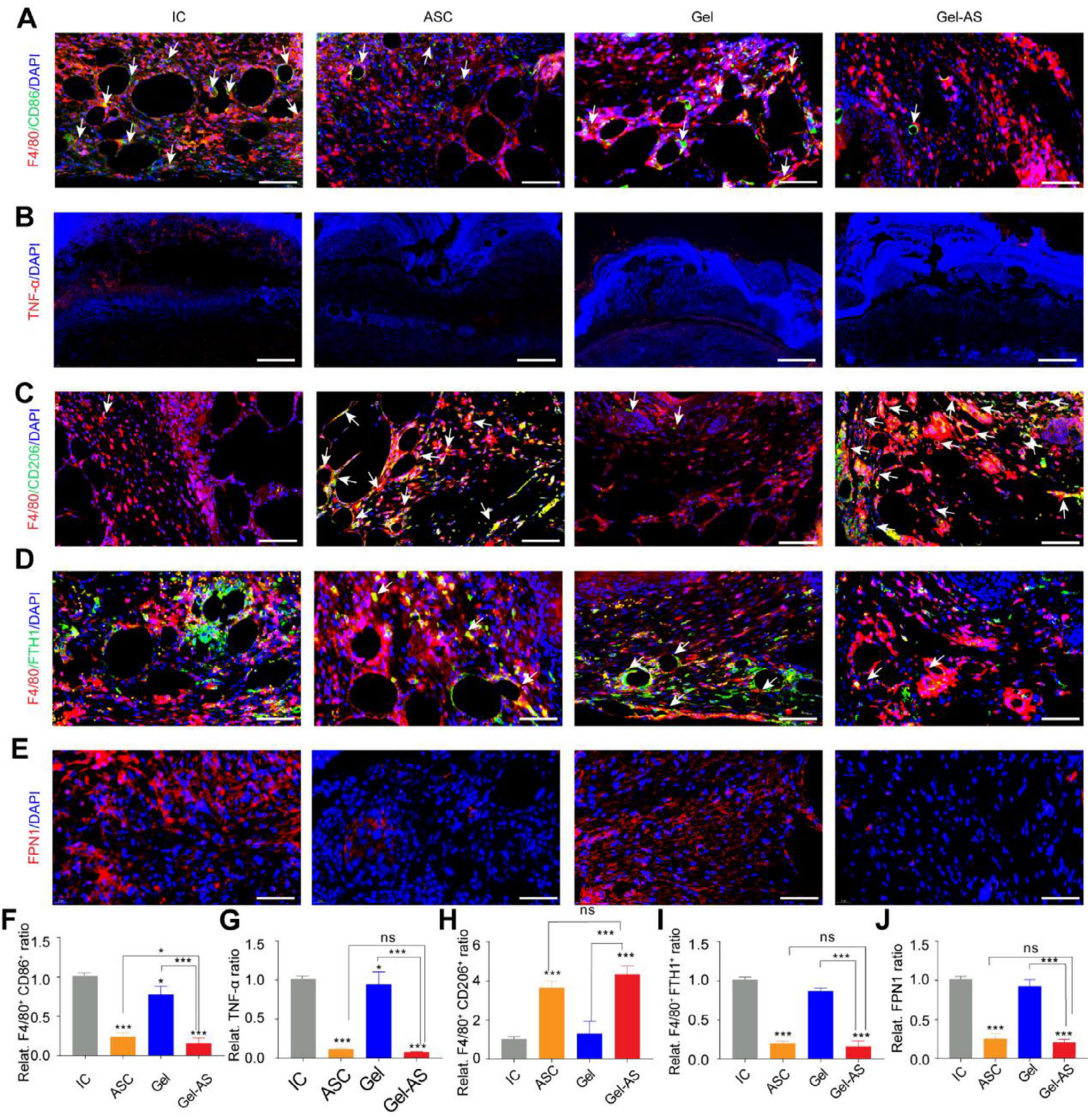
Gel‐AS activated ferroptosis of M1 macrophages in vivo. (A) Immunofluorescent staining images of F4/80^+^CD86^+^ cells (indicated by white arrows) at wounds treated with IC, ASC, Gel, and Gel‐AS on D7. Scale bar, 200 µm. (B) Immunofluorescence staining of TNF‐α^+^ cells at wounds with different treatments on D7. Scale bar, 500 µm. (C) Immunofluorescent staining images of F4/80^+^CD206^+^ cells (white arrows) at wounds with different treatments on D7. Scale bar, 200 µm. (D) Immunofluorescence staining of F4/80^+^ FTH1^+^ cells (white arrows) at wounds with different treatments on D7. Scale bar, 200 µm. (E) Immunofluorescence staining of FPN1 at wounds with different treatments on D7. Cell nucleus was dyed with DAPI (blue fluorescence). Scale bar, 200 µm. (F) Corresponding quantitative statistics of F4/80^+^CD86^+^ cells in (A) (mean ± SD; one‐way ANOVA, *n* = 6). (G) Quantitative analysis of TNF‐α in (B) (mean ± SD; one‐way ANOVA, *n* = 6). (H) Corresponding quantitative statistics of F4/80^+^CD206^+^ cells in (C) (mean ± SD; one‐way ANOVA, *n* = 6). (I) Quantitative analysis of FTH1 in (D) (mean ± SD; one‐way ANOVA, *n* = 6). (J) Quantitative analysis of FPN1 in (E) (mean ± SD; one‐way ANOVA, *n* = 6). Statistically significant differences between groups are indicated as follows: ns, not significant, * p < 0.05 and ***p < 0.001.

However, the number of M1 macrophages in wounds treated by ASC and Gel‐AS was significantly lower than that in the other two groups on D7 (Figure 8A,F). The number of M1 macrophages in the Gel‐AS‐treated wounds was the lowest. Furthermore, the secretion of TNF‐α was markedly diminished in the ASC and Gel‐AS groups on D7 (Figure 8B,G). The number of M2 macrophages in the Gel‐AS‐treated group was significantly higher than that in the other three groups (Figure 8C,H). Therefore, AS did not delay the activation of skin‐related functional cells, and Gel‐AS‐treated wounds had already progressed from the inflammatory phase to the proliferative phase on D7, while the other groups remained in the inflammatory phase. This result further verified that Gel‐AS could propel healing process of diabetic wounds from inflammatory phase to proliferative phase quickly. We also investigated the expression of FTH1 and FPN1 proteins at wound sites on D7. The expression levels of FTH1 (Figure 8D,I) and FPN1 (Figure 8E,J) were lowest in Gel‐AS treated wounds, further verifying that AS activated ferroptosis of M1 macrophages in vivo. Collectively, these results indicate that the sustained release of AS from Gel induced ferritinophagy to activate ferroptosis of M1 macrophages in vivo, thereby shifting healing process from inflammatory phase to the proliferative phase.

## 3 Discussion

DFUs are a major complication of diabetes mellitus, accounting for 80% of lower extremity amputations and increasing the risk of mortality in diabetic patients [42]. Pathologically, the main factor impeding the healing of diabetic wounds has been identified as the persistent inflammation, mediated by the upregulated pro‐inflammatory cytokines, the excessive accumulation of M1 macrophages, and the blocked M2 macrophage polarization [4, 42], Reportedly, macrophages were more active at diabetic wounds and prone to be polarized into the proinflammatory M1 phenotype compared with healthy ones [43]. There are several possible cues. First, the persistent HG microenvironment directly upregulates the expression levels of proinflammatory cytokines and promotes their secretion by increasing the activities of RhoA/ROCK of M1 macrophages [44] and altering histone acetylation and methylation levels in surrounding cells [45, 46]. Second, in HG microenvironment, macrophages become sensitized to the stimuli of cytokines and their glycolytic capacities are reduced, impairing their phagocytosis and bactericidal abilities [12]. Third, Gram‐negative bacteria infection was reported to account for approximately 83.7% of infections in chronic wounds, leading to the excessive accumulation of LPS [47, 48, 49]. Collectively, these cues synergistically result in the excessive accumulation of M1 macrophages at diabetic wounds. Therefore, reducing the number of M1 macrophages and promoting macrophage polarization are important strategies to resolve the persistent inflammatory state [50, 51, 52]. Reportedly, some signal pathways and receptors were claimed to induce macrophage polarization. The MAPK and NF‐κB signaling pathways significantly influenced the secretion of inflammatory cytokines and the viability of macrophages [53]. Furthermore, the TLR4 receptor on macrophages could control the secretion of inflammatory cytokines via regulating NF‐κB signaling pathway, thereby preventing LPS‐induced excessive inflammation and inducing macrophage polarization [54]. However, how to reduce the number of M1 macrophages has not been discussed thoroughly.

Ferroptosis, as a novel form of regulatory cell death, is regulated by several metabolic signaling pathways, including oxidative‐redox‐metabolism mediated by SLC7A11, GSS or GCLC, FTH1‐dominated iron‐metabolism, and LPCAT3‐regulated lipid‐metabolism [55]. In fact, a bundle of literatures has revealed that ferroptosis directly mediates the immune response soon after ferroptosis was reported in 2012 [56]. Interestingly, it is reported that the immune activity of M1 macrophages was limited by the occurrence of iron‐overload‐related ferroptosis during the clearance of aged red blood cells [57]. Additionally, M1 macrophages can kill pathogenic bacteria through the generation of ROS, which can lead to the occurrence of oxidative‐redox‐related ferroptosis within M1 macrophages [58]. Therefore, we speculated that activating ferroptosis in M1 macrophages might be a strategy to reduce the excessive accumulation of M1 macrophages at diabetic wound sites.

Many clinical studies have confirmed the role of *Centella asiatica* in promoting the healing process of diabetic wounds. For example, Huang et al. compared the therapeutic outcomes between topical application of ON101 cream (containing one active ingredient of the extract of *Centella asiatica*) and standard care in randomized phase 3 clinical trial of 236 patients with DFUs. They reported that the topical application of ON101 with gauze immediately following debridement showed notable healing efficacy in all patients by regulating macrophage polarization compared with an absorbent dressing [24]. Previous studies have shown that AS can inhibit the NF‐κB signaling pathway in LPS‐stimulated RAW264.7 cells to downregulate the secretion of pro‐inflammatory cytokines [28, 29]. However, the immune‐regulatory properties of AS on macrophage behavior in HG microenvironment and the underlying mechanisms remain an unsolved mystery.

In this study, we systematically investigated the molecular mechanisms behind the anti‐inflammatory function of AS on M1 macrophages and developed an anti‐inflammatory wound dressing by encapsulating AS into biocompatible GelMA. We observed that AS inhibited the proliferation and secretion of pro‐inflammatory cytokines from HG‐M1 macrophages and LPS‐M1 macrophages, and reduced the number of M1 macrophages significantly. This unique phenomenon inspired us to deeply explore the secrets behind the anti‐inflammatory secrets of AS on M1 macrophages.

Notably, a previous study reported that iNOS reduced the binding ability of RSL3 to GPX4, which reduced the sensitivity of M1 macrophages to RSL3 (the ferroptosis activator) [35]. However, in our experiment, we observed ferroptosis occurred in HG‐M1 macrophages and LPS‐M1, instead of IL‐4‐M2 macrophages, after the treatment with AS. Therefore, we further investigated ferroptosis‐associated characteristics in HG‐M1 macrophages, including the accumulation of free Fe^2+^ and ROS levels, the morphology changes of mitochondria, and the expression levels of ferroptosis‐related genes and proteins, systematically. These results indicated that AS indeed activated ferroptosis inside HG‐M1 macrophages. Next, the rescue experiments of DFO and Erastin were used to further clarify the crucial role of AS‐mediated ferroptosis in inhibiting the immune activities of HG‐M1 macrophages. DFO is an iron chelator that effectively absorbs free Fe^2+^ to reduce ROS level in cells, reducing the consumption of GPX4, and inhibiting ferroptosis. While, Erastin can induce cellular ferroptosis by binding SLC7A11 and inhibiting its expression, which in turn suppresses the activity of GPX4 to activate ferroptosis [59]. Clearly, the rescue results showed that the AS‐mediated ferroptosis in HG‐M1 macrophages was antagonized and enhanced by the pretreatment of DFO and Erastin, respectively, demonstrating that AS exerted immunomodulatory properties on HG‐M1 macrophages by activating ferroptosis.

Furthermore, the abnormal regulatory behavior of AS on the expression levels of FTH1 gene and protein suggested that AS could lead to the degradation of FTH1 protein in HG‐M1 macrophages. FTH1 is an intracellular iron‐storage protein that ensures secure binding of free Fe^2+^ and thereby serves as an additional antioxidant [60]. Reportedly, FTH1 protein can be transferred to and degraded by ALs by binding to NCOA4, which is referred to as ferritinophagy [36]. In our experiment, we observed that AS upregulated the expression levels of NCOA4 protein and autophagy‐associated proteins in a dose‐dependent manner. Furthermore, co‐localization of FTH1 and LAMP2, and tandem fluorescent Ad‐mRFP‐GFP‐LC3 (tf‐LC3) probe assay suggested the activation of ALs in HG‐M1 macrophages with the treatment of AS. Meanwhile, the rescue experiments with 3‐MA and RAPA were used to indicate the crucial role of ferritinophagy in AS‐mediated ferroptosis in HG‐M1 macrophages. 3‐MA is a commonly used autophagy inhibitor that targets class III phosphatidylinositol 3‐kinase complex (PI3KC3) to prevent the formation of the PIK3C3 complex [59]. RAPA is an autophagy activator that can block the mTOR signaling pathway [60]. Clearly, ferritinophagy in AS‐mediated ferroptosis was antagonized and enhanced by the pretreatment with 3‐MA and RAPA, respectively, demonstrating that AS exerted immunomodulatory properties on HG‐M1 macrophages by activating ferritinophagy‐mediated ferroptosis.

Another interesting phenomenon that the lower expression levels of FPN1 protein in HG‐M1 macrophages gave us further inspiration that AS could regulate the expression of FPN1 protein to further activate ferroptosis. The rescue experiments to overexpress or silence the expression of the FPN1 gene in HG‐M1 macrophages showed that the FPN1 protein mediated AS‐activated ferroptosis in HG‐M1 macrophages by blocking the transport of free Fe^2+^ out of the cells. Overall, AS disrupted the iron‐metabolism to activate ferroptosis in M1 macrophages by the orchestrated collaboration of ferritinophagy and downregulation of FPN1 protein expression. These results indicated that developing AS‐based anti‐inflammatory dressings to target ferroptosis of M1 macrophages could be a promising strategy to alleviate persistent inflammation at diabetic wounds.

Regrettably, lower water‐ or oil‐solubility and limited ionization of AS restricted its bioavailability and hindered potential applications [21]^.^ Numerous biomaterials have been used as drug delivery vehicles, such as nanozymes [18], liposomes [61], metal−organic framework [62], microspheres [21], and hydrogel patch [13, 14, 15]. As a kind of biocompatible hydrogels, Gels have been recognized as attractive tissue engineering materials in regenerative medicine and wound healing to achieve the sustained release of bioactive drugs and provide moist microenvironment [63]. Their porosity, mechanical, swelling, and degradation properties can be easily tailored by changing the degree of cross‐linking [64]. Additionally, the pore size of Gel could affect the release kinetics of AS and influence the polarization of macrophages by regulating the mechanical stiffness [65, 66]. Bigger pore size often leads to quicker releasing rate of AS, while smaller pore size results in slower releasing rate. Furthermore, bigger pore size leads to lower mechanical stiffness to promote the expression of anti‐inflammatory cytokines. On the contrary, smaller pore size leads to higher mechanical stiffness to promote the expression of pro‐inflammatory cytokines. In this study, the pore size and the Young modulus of 10%‐Gel was approximately 75 µm and 7.4 kPa, which showing potential to stimulate macrophage polarization toward M2 subtype. In addition, AS was completely released from 10%‐Gel on D8, thereby matching inflammatory stage of the healing process. Therefore, 10%‐Gel was subsequently selected to carry AS in future experiments.

Then, we used Gel‐AS to treat full‐thickness diabetic wounds in rodent models, taking IC, ASC, and Gel as controls. The wounds treated with Gel‐AS showed the fastest healing rate compared with the other three groups. Furthermore, the number of F4/80^+^CD86^+^ and F4/80^+^CD206^+^ cells in the Gel‐AS‐treated wounds on D7 was the lowest and the highest, respectively, indicating that the Gel‐AS‐treated wounds had already passed the inflammatory phase to the proliferative phase on D7. Meanwhile, the expression levels of FTH1 and FPN1 proteins were lowest in Gel‐AS‐treated wounds, further confirming that AS activated ferroptosis of M1 macrophages in vivo.

In conclusion, we systematically revealed the underlying molecular mechanisms behind the anti‐inflammatory secrets of AS on M1 macrophages. Briefly, AS disrupted the iron metabolism to activate ferroptosis in M1 macrophages in vitro and in vivo. Specifically, AS activated the ferritinophagy process, resulting in the degradation of FTH1 by ALs, followed by the release of massive free Fe^2+^ in M1 macrophages. Meanwhile, AS downregulated the expression of FPN1 to prevent the transportation of free Fe^2+^ to out of the cells. The orchestrated cooperation between these two AS‐mediated processes disrupted the iron‐metabolism to activate ferroptosis inside M1 macrophages. Moreover, we loaded AS into Gel to produce an anti‐inflammatory wound dressing (Gel‐AS). Convincingly, Gel‐AS inhibited the expression of FPN1 and FTH1 proteins on D7 at diabetic wounds, shortening the inflammatory phase and promoting the healing process of diabetic wounds. We believe that unraveling the mysterious veil of AS to activate ferroptosis in innate immune cells could be a new therapeutic target for inflammation‐associated diseases.

## 4 Experimental Section

### 4.1 Materials

Fetal bovine serum (FBS, 12483020), Dulbecco's modified eagle medium (DMEM, 11960044), opti‐modified eagle's medium (Opti‐MEM, 11058021), and penicillin‐streptomycin solution (PS, 15140122) were obtained from Gibco (USA). Lipofectamine 2000 (11668019) and FPN1 (PA5‐115915) were procured from Invitrogen (USA). AS (RFS‐J00101910008, the purity was greater than 98%) was sourced from RuiFenSi Biotechnology Co. (China). Phosphate buffered solution (PBS, P1010), ethylene diamine tetra‐acetic acid (EDTA, E8030) solution, H&E (G1120), cell counting kit‐8 (CCK‐8, CK04), radio immunoprecipitation assay lysis buffer (RIPA lysates, R0010), bicinchoninic acid protein assay kit (BCA, PC0020), ECL plus ultra‐sensitive kit (PE0010), 5% bovine serum albumin (BSA) blocking buffer, and Hoechst 33342 (C0031) were purchased from Solarbio (China). 5‐Ethynyl‐2‐deoxyuridine (EdU, C0078S) kit, quickblock western primary antibody dilution buffer (P0256) and polyvinylidene fluoride (PVDF, FFP36) membrane were from Beyotime (China). TNF‐α (D2D4) XP rabbit mAb (11948), CD11c (N418) hamster mAb (PE conjugate, 73359), and LC3B (4108S) were sourced from Cell Signaling Technology (CST, USA). Autophagy‐related molecules such as ATG5 (A0203), ATG7 (A21895), p62 (A19700), ferroptosis‐related molecules FTH1 (A19544), GPX4 (A11243), SLC7A11 (A2413), and macrophage polarization‐related factor CD86 (A1199) were obtained from Abclonal (China). NCOA4 (PA5‐96398), iNOS (18985‐1‐AP), IL‐1β (16806‐1‐AP), and LAMP2 (66301‐1‐Ig) were purchased from Proteintech (USA). Macrophage polarization‐related factors F4/80 (ab6640) and CD206 (ab300621) were procured from Abcam (UK). GelMA (EFL‐GM‐PR) was sourced from Engineering for Life (China), while IC (000418) came from YIHAN Biology (China). RNA isolation kit (ES‐RN001) was obtained from YI SHAN (China). SYBR Green Premix Pro Taq HS qPCR Kit (ROX Plus, AG11718), Microseal PCR plate sealing film (AG12106), and qPCR 96‐well plates (AG12102) were sourced from Accurate Biology (China). PAGE gel fast preparation kit (D032202 and D032203) was procured from Yangguang Biology (China). Alexa Fluor 594‐conjugated goat anti‐rabbit immunoglobulin G (IgG; H+L) antibody (A‐11012), Alexa Fluor 594‐conjugated donkey anti‐mouse IgG (H+L) antibody (ZF‐0516), anti‐mouse IgG (H+L) (ZF‐0513), Alexa Fluor 488‐conjugated goat anti‐rabbit IgG (H+L) antibody (ZF‐0511), horseradish peroxidase (HRP)‐labeled goat anti‐rabbit IgG (H+L, ZB‐2306) antibody, and HRP‐labeled anti‐mouse IgG antibody (H+L, A0216) were all obtained from Zhongshan Golden Bridge Biotechnology (China). Ferro‐orange was procured from DojinDo (Japan). 3‐MA (AY‐22989), RAPA (S2767), Erastin (S7242) and DFO (Ba33112) were sourced from Selleck (USA). FITC rat anti‐mouse CD86 (561962) came from BD Biosciences (USA). Triton X‐100 (T8787) was obtained from Sigma (Germany). LPS (HY‐D1056) was procured from MCE (USA), while siRNAs, FPN1 OE and NC came from Tsingke Biotechnology (China). The GPX4 (JK‐TY‐G55236), GSS (JK‐HU‐G1558), LPCAT3 (JK‐HU‐L71525) assay kits were obtained from Jingkey (China). The DHE‐ROS kit (BB‐47051) was sourced from BestBio (China) and the Ad‐mRFP‐GFP‐LC3 adenovirus (HB‐AP2100001) was obtained from Hanheng Biotechnology Company (China). All reagents were used as received without any further treatment.

### 4.2 Cell Culture

RAW264.7 cells (TIB‐71, USA) were obtained from the American type culture collection (ATCC). The RAW264.7 cells were cultured in DMEM supplemented with 10% FBS and 1% PS, alongside different final concentrations of glucose (25 mM for NG and 50 mM for HG) for 60 days to obtain NG‐RAW264.7 cell (NG macrophages) and HG‐RAW264.7 cells (HG‐M1 macrophages). For LPS stimulus, NG‐RAW264.7 cells were cultivated in a DMEM medium with LPS (100 ng/mL) for 12 h to obtain LPS‐M1 macrophages.

### 4.3 Transient Transfection

To investigate the potential function of FPN1 protein in AS‐induced ferroptosis, transfection reagents were used to treat HG‐M1 macrophages cells. Specifically, 500 µL of Opti‐MEM, 5 µL of Lipofectamine 2000, and 5 µL of siFPN1‐1,2,3 (The FPN1‐siRNA sequences were as follows: siRNA‐1 GATGGGTCTCCTACTATAA, siRNA‐2 GACATGAATGCTACCATTA, and siRNA‐3 GGGTCCTTACTGTCTGCTA), FPN1 OE or FPN1 NC were added to HG‐M1 macrophages cells and incubated for 12 h. The measurement and evaluation of transfection efficiency were carried out using qRT‐PCR and Western blotting analysis.

### 4.4 CCK‐8 Assay

For the CCK‐8 assay, RAW264.7 cells were seeded in 96‐well plates and subjected to various treatments. The effect of glucose was examined by cultivating and passing the cells in NG‐DMEM or HG‐DMEM over 60 days. To study the impact of AS, AS powder was dissolved in DMSO to prepare the stock solution (100 mM). Then, this stock solution was diluted with DMEM to obtain AS with different concentrations (AS1 for 0.001 mM, AS2 for 0.01 mM, and AS3 for 0.1 mM). Subsequently, HG‐M1 macrophages were cultured in medium containing AS1, AS2, or AS3. To explore the way that AS induced the programmed cell death of HG‐M1 macrophages, HG‐M1 macrophages were pre‐exposed to ZVD, NEC‐1, 3‐MA, DFO, RAPA, or Erastin, respectively, with specifical concentration and time before receiving AS3 treatment. To evaluate the critical role of FPN1 in determining cell viability of HG‐M1 macrophages, HG‐M1 macrophages were pre‐transfected with transfection reagents containing FPN1‐siRNAs, FPN1‐OE, or FPN1‐NC plasmids for 12 h. To assess the influence of FPN1 protein on mediating AS‐inhibited cell viability of HG‐M1 macrophages, HG‐M1 macrophages were pre‐transfected with siFPN1s, FPN1‐OE or FPN1‐NC plasmid, and underwent the treatment of AS3. After treatments, each well was supplemented with 10 µL of the CCK‐8 kit and incubated for another 2 h before the measurement of absorbance at 450 nm using a microplate reader (1201‐7044, Thermo fisher scientific, USA).

### 4.5 EdU Assay

The EdU assay was conducted according to the specified guidelines from the manufacturer. RAW264.7 cells were in 24‐well plates and suffered different treatment. To scrutinize the effect of AS on the proliferation behavior of RAW264.7 cells, HG‐M1 macrophages, and LPS‐RAW264.7 cells were cultured in DMEM containing AS3, taking NG macrophages as the control. To assess the influence of autophagy on proliferation behaviors of HG‐M1 macrophages, HG‐M1 macrophages were pre‐treated with 3‐MA or RAPA, and underwent treatment with AS3. To assess the influence of FPN1 protein on proliferation behaviors of HG‐M1 macrophages, HG‐M1 macrophages were transfected with siFPN1s, FPN1 OE plasmid, FPN1 NC plasmid. To assess the influence of FPN1 protein on mediating AS‐inhibited proliferation behaviors of HG‐M1 macrophages, HG‐M1 macrophages were pre‐transfected with siFPN1s, FPN1 OE plasmid, FPN1 NC plasmid, and underwent the treatment of AS3. Then, EdU reagent diluted in DMEM (1:1000, v/v) was added to 24‐well plates and incubated at 37°C for 12 h. Next, the cells were fixed with paraformaldehyde for 15 min and permeabilized for 10 min. After that, EdU reaction solution (exhibiting red fluorescence) were added and co‐cultivated for 30 min. EdU positive cells were imaged using a fluorescence microscope (EVOS5000, Invitrogen, USA).

### 4.6 FCM Assay

For the proportions of cells in HG‐/LPS‐M1 macrophages: FCM was used for the proportions of CD11c^+^CD86^+^ cells in HG‐/LPS‐M1 macrophages. To assess the impact of HG on macrophage polarization, the proportions of CD11c^+^CD86^+^ cells in RAW264.7 cells and HG‐M1 macrophages was analyzed using FCM. To evaluate the influence of AS and FPN1 on macrophage polarization, the HG‐M1 macrophages were co‐cultured in a medium contained various concentrations of AS (either independently or in combination with siFPN1or FPN1 OE). The variation in the proportion of CD11c^+^CD86^+^ cells due to different AS concentration was further investigated by culturing RAW264.7 cells in a medium containing with 100 ng mL^−1^ LPS (either standalone or combined with AS3). Meanwhile, the proportion of CD163^+^ cells in LPS‐M1 macrophages were analyzed after treating with various concentrations of AS. The impact of varying AS concentrations on the proportion of CD163^+^CD206^+^ macrophages was examined in IL‐4 pre‐treated RAW264.7 cells (20 ng mL^−1^, 24 h). Following the co‐culture period, cells were subjected to trypsinization, washed twice with PBS, and separated into tubes with the density of 1 × 10^6^ cells per tube. Prior to FCM analysis, Fc receptors were blocked by pre‐incubating cells with a purified blocking reagent for 10 min on ice. Separate staining procedures were then conducted using antibodies. The final concentration of antibodies reached 1%. Blank controls consisted of cells that were not treated with antibodies. The cell suspension incorporating the antibodies was incubated on ice in the dark for 30 min and then washed twice with PBS. Cells stained positively were detected using a BD FACSCelesta instrument (BD Biosciences, USA).

### 4.7 ROS Level of HG‐M1 Macrophages

HG‐M1 macrophages were cultured in DMEM containing AS1, AS2, or AS3. Then, these cells were subjected to staining using the PE‐ROS kit at 37°C for 30 min. Detection of positively stained cells was subsequently executed utilizing a BD FACSCelesta instrument (BD Biosciences, USA).

### 4.8 ELISA Assay

To explore the effect of AS on the enzyme catalytic activities of GPX4, GSS, and LPCAT3 of HG‐M1 macrophages cells. The HG‐M1 macrophages cells were pretreated with AS1, AS2, or AS3 for 24 h, taking NG‐RAW264.7 cells as the control. Subsequently, the cells were centrifugated at 3000 rpm for 30 min, after which the supernatant was removed. Then, the serially diluted standard solution (40 µL) and samples (10 µL) were added to the ELISA plate wells and incubated with horseradish peroxidase‐conjugated specific antibodies for GPX4, GSS and LPCAT3 for 60 min at 37°C. Next, the ELISA plate wells were washed five times with washing solution. After that, 50 µL of the supplied substrate A and 50 µL of B in the kit were added to each well and the specimens were incubated at 37°C in the dark for 15 min. Ultimately, the absorbance value at 450 nm was measured (1201‐7044, Thermo fisher scientific, USA).

### 4.9 Immunofluorescence Staining

To investigate the expression of TNF‐α, iNOS and IL‐1β in RAW264.7 cells after different treatments, immunofluorescence staining was performed. Additionally, the colocalization of FTH1 and TNF‐α or LAMP2 in RAW264.7 cells was also examined. After 24 h of treatment, RAW264.7 cells were fixed, permeabilized with 0.1% Triton X‐100, and blocked with 2% BSA. Subsequently, the cells were incubated overnight with TNF‐α primary antibodies (diluted 1:200), a combination of FTH1 (1:200) and TNF‐α (1:200), LAMP2 (1:200), iNOS (1:200) or IL‐1β (1:200) primary antibodies. Following this, a secondary antibody was applied. The immunofluorescence staining was visualized using a fluorescence microscope (EVOS5000, Invitrogen, USA).

### 4.10 qRT‐PCR Analysis

To explore the effect of HG on the expression levels of proinflammatory genes including TNF‐α, iNOS, and IL‐1β, RAW264.7 cells were cultured and passaged in NG‐DMEM or HG‐DMEM for 60 days. To study the effect of AS on the expression levels of proinflammatory genes including TNF‐α, iNOS, and IL‐1β, and ferroptosis‐related genes expression levels of SLC7A11, GCLC, LPCAT3, GSS, FTH1, FPN1, and GPX4 genes, HG‐M1 macrophages were co‐cultured with AS1, AS2, or AS3 for 24 h. To assess the levels of autophagy‐related genes including ATG5, ATG7, p62, and LC3B, HG‐M1 macrophages were subjected to AS with different concentrations (either the presence or absence of RAPA or 3‐MA). Then, RNA extraction was carried out using an RNA Isolation Kit obtained from YI SHAN Biotechnology. Briefly, spin columns filled with the cell‐lysis solution were subjected to centrifugation at 12,000 g for 1 min. Then, the spin columns were washed with the washing buffer and centrifuged again at 12,000 g for 1 min. Subsequently, the columns were transferred to new EP tubes and diluted with DEPC water, followed by centrifugation at 12,000 g for 1 min. SYBR Green PCR Master Mix (Thermo Fisher Scientific, USA) was used for qRT‐PCR on a 7500HT fast real‐time PCR System (Thermo fisher scientific, USA). The primer sequences utilized in the experiment are listed in Table S1.

### 4.11 Western Blotting Assay

To quantify expression levels of ferroptosis‐related proteins including SLC7A11, FTH1, GPX4, HG‐M1 macrophages were co‐cultured with AS1, AS2, and AS3 for 48 h. Meanwhile, the expression level of GPX4 protein in DFO or Erastin‐pretreated HG‐M1 macrophages with post‐treatment of AS were also detected. To measure the expression levels of autophagy‐related proteins including ATG5, ATG7, p62, and LC3II/LC3I, HG‐M1 macrophages were co‐cultured with AS1, AS2, and AS3, taking NG‐RAW264.7 cells, and HG‐M1 macrophages as controls. To study the role of ferroautophagy role in AS‐mediated ferroptosis, the expression levels of SLC7A11, FTH1, and GPX4, HG‐M1 macrophages were pre‐treated with RAPA or 3‐MA, followed by the post‐treatment of AS3. For detecting the expression level of FPN1 proteins, HG‐M1 macrophages were co‐cultured with AS1, AS2, AS3, taking NG‐RAW264.7cells, HG‐M1 macrophages as controls. To demonstrate the silencing or overexpression efficacy of siFPN1s, FPN1 OE plasmid or FPN1 NC plasmid, HG‐M1 macrophages were transfected with siFPN1s, FPN1 OE plasmid or FPN1 NC plasmid. To clarify the downregulating role of AS on FPN1 protein, HG‐M1 macrophages were transfected with siFPN1, FPN1 OE plasmid or FPN1 NC plasmid and then cultured in DMEM containing AS3. Then, RIPA lysates were utilized to extract proteins in a brief procedure. The protein content was quantified using a BCA kit. Electrophoresis was then conducted for 90 min at 120 V. Following electrophoresis, the proteins were transferred onto PVDF membranes through electroblotting and subsequently blocked with 5% milk for 1 h. PVDF membranes were then co‐incubated with primary antibodies at a dilution of 1:1000, followed by secondary antibodies at a concentration of 1:10,000. Antibody reactivity was detected using an ECL kit (Solarbio, China) and visualized with the UVITEC alliance mini HD9 system (Britain). The gray value, representing protein expression level, was quantified using Image J software.

### 4.12 Autophagic Flux in RAW264.7 Cells

In the preliminary step, NG‐RAW264.7 cells, HG–RAW264.7 cells, AS3‐treated HG‐M1 macrophages cells, 3‐MA or RAPA‐pre‐treated HG–RAW264.7 cells with the post‐treatment of AS3 were cultured on Confocal dish and infected with Ad‐mRFP‐GFP‐LC3b following the manufacturer's instructions. The virus transfection was conducted at a multiplicity of infection (MOI) of 50 for 6 h. Thereafter, the adenovirus‐containing supernatant was removed, and the cells were incubated in fresh medium for another 24 h. The treated cells were washed with PBS. ALs were represented by red spots, while yellow spots indicated APs. Images were acquired using Confocal laser microscopy (Nikon C1 laser scanning confocal, Japan).

### 4.13 TEM Analysis of the APs, ALs, and Mitochondrial Ultrastructure

First, RAW264.7 cells were treated with NG, HG, or HG+AS3. Subsequently, the treated cells were fixed with 2.5% glutaraldehyde for 4 h and washed twice with PBS. To facilitate further analysis, the cells underwent gradient dehydration. Samples were then embedded in epoxy resin and sliced with the thickness of 90 nm accordingly. Finally, image observation was conducted using the TEM (Hitachi, Japan).

### 4.14 Detecting Free Fe^2+^ and ROS

For investigating the intracellular free Fe^2+^ and ROS, RAW264.7 cells were subjected to treatments identical to above. After the treatments, RAW264.7 cells were exposed with 10 µM Ferro‐orange (the indicator for Fe^2+^) or DHE (the indicator for ROS) for 30 min. Subsequently, the cell nuclei were stained by Hoechst 33342 dye. Images were captured using a fluorescence microscope (EVOS5000, Invitrogen, USA).

### 4.15 Detecting Intracellular GSH Levels

To measure intracellular GSH levels, a GSH assay kit was employed under the manufacturer's instructions. RAW264.7 cells were co‐cultured under NG, HG, or HG+AS3 conditions for 24 h. After rinsing with PBS, the cells were incubated and subsequently centrifuged (4°C, 1000 rpm) to obtain the cell precipitate. The cell precipitate was then treated with protein removal reagent (M solution). To facilitate GSH extraction, the mixture suffered from three cycles of freeze‐thaw, followed by extraction of the supernatant through centrifugation. The absorbance of each sample was measured at 412 nm using a spectrophotometer (1201‐7044, Thermo fisher scientific, USA). Cellular GSH content was calculated based on triplicate measurements using standard curves generated in parallel.

### 4.16 Characterization of Gel

The morphology and mechanical strength of Gels were characterized via SEM and a universal testing machine (CMT6103, China). For the SEM analysis, Gels with different monomer concentration were cured under UV‐light (*λ* = 365 nm) for 10 s at room temperature (RT). Then, these Gels were refrigerated before undergoing lyophilization under a condensing temperature of −50°C and vacuum degree of 10 Pa for 48 h. The morphology of the Gels was observed using SEM (300, Sigma, USA). Fourier transform infrared spectroscopy (FTIR) and X‐ray photoelectron spectroscopy (XPS) were used to detect the chemical structure and elemental composition of Gel and Gel‐AS. Parameters such as pore size and porosity were calculated using Image J software. The stress–strain curve of the Gels was generated on a CMT6103 universal testing machine with the tensile speed of 10 mm/min (Sans, China) during the testing process. Additionally, swelling experiments were conducted by immersing Gels in an excess of deionized water maintained at 25°C. Then, the weight of Gels at different timepoints were recorded. The swelling rate was calculated according to Equation (1). To obtain the degradation rate of Gels, Gels were immersed in DPBS containing type II collagenase (0.02 U mL^−1^) at 37°C. Then, the weight of Gels at different timepoints were recorded. The degradation rate was calculated according to Equation (2).

(1) $$\mathrm{Swelling}\;\mathrm{rate}\;\left(\%\right)=\frac{W_{2}-\;W_{1}}{W_{1}}\;\times 100\%$$

*W*_1_: The initial weight of Gel, *W*_2_: The weight of Gel at a different time.

(2) $$\mathrm{Degradation}\;\mathrm{rate}\;\left(Wx\%\right)=\frac{W_{1}-\left(W_{1}-W_{2}\right)}{W_{1}}\times 100\%$$

*W*_1_: the initial weight of Gel, *W*_2_: the weight of Gel at a different time, and *W_X_*: the weight remained.

### 4.17 Release Ratio of AS From Gels In Vitro

The mixture of Gel and AS was prepared by adding 0.078 mL of AS into 1.422 mL of Gels. Then, the mixed solution was divided into 14 droplets (each containing 0.1 mL) and cured for 10 s under UV‐light (*λ* = 365 nm) at RT. The prepared Gel‐AS was placed in the upper compartment of a Transwell chamber, while 1 mL of PBS was positioned in the lower compartment. The PBS containing released AS was collected on D0, D2, D4, D6, D8, D10, D12, and D14, and the amount of AS in the collected PBS was quantified using HPLC (Agilent 1260 series, USA).

### 4.18 In‐Vitro Biocompatibility of Gel and Gel‐AS

Calcein acetoxymethyl ester‐propidium iodide (calcein‐AM/PI) double staining was employed to access the biocompatibility of Gel‐IS and Gel‐AS‐IS on HG‐HUVECs and HG‐HDFs. HG‐HUVECs and HG‐HDFs were seeded in 24‐well plates (5 × 10^3^ cell mL^−1^) and incubated for 24 h. Then, Gel‐IS, Gel‐AS‐IS, or PBS (100 µL) was added to the culture medium of the cells and incubated for another 24 h. Next, the calcein‐AM/PI reagents were added to the cells with different treatments and incubated for 30 min in the dark according to the manufacturer's protocol. The fluorescence intensity was visualized using a fluorescence microscope.

### 4.19 The In Vivo Biocompatibility of Gel‐AS

24 male DB/db mice (7 weeks old) were used to assess the biocompatibility of Gel and Gel‐AS in vivo (*n* = 6). The mice were anesthetized with pentobarbital sodium at a dosage of 50 mg kg^−1^ and shaved. Subsequently, the full‐thickness skin wounds with a diameter of about 1 cm were created on their backs using a biopsy punch. Then, the mice were divided into four groups randomly (*n* = 6) and treated with IC, ASC, Gel, and Gel‐AS. The major organs (liver, heart, spleen, lung, and kidney) of mice were collected on D14 after surgery and analyzed by H&E staining.

### 4.20 Wounding and Treating Procedure of Skin Defect Model in Mice

24 male DB/db mice (7 weeks old) were acquired from View solid Biotechnology (Beijing, China). All animal experiments received approval and guidance from the Institutional Animal Care and Use Committee at View Solid Biotechnology (No. vs2126A00724). The mice were housed under standard experimental animal feeding conditions. After a 1‐week adaptive feeding period, they were anesthetized with pentobarbital sodium at a dosage of 50 mg kg^−1^ and shaved. Using a biopsy punch, full‐thickness skin wounds (diameter of about 1 cm) were then created on both sides of their backs. Subsequently, the mice were randomly divided into four groups: IC, ASC, Gel, and Gel‐AS (*n* = 6). The IC was supplemented with 10.4 µL of DMSO to obtain the IC group. The IC was supplemented with 10.4 µL of DMSO containing 100 mM AS to fabricate the ASC group. Then, the IC and ASC groups were stirred thoroughly using a magnetic stirrer and applied to the wounds (3.5 mg, twice daily) on D0, D3, and D6. For the Gel group, 10.4 µL of DMSO was added to 1989.6 µL of 10%‐Gel. For the Gel‐AS group, 10.4 µL of 100 mM AS was incorporated into 1989.6 µL of 10%‐Gel. A volume of 100 µL sample was applied to the diabetic wound at D0 in Gel and Gel‐AS groups. The healing process was monitored by capturing images of the wounds on D0, D3, D7, and D14, respectively.

### 4.21 Histological Analysis

Examination of edge width in wound tissues of IC, ASC, Gel or Gel‐AS groups at D7 was conducted using H&E staining. After the mice were euthanized, wound tissue samples were carefully excised and then preserved with a solution of 4% paraformaldehyde. These tissues were subsequently embedded in paraffin and sectioned into 4 µm thick slices using a paraffin slicing machine (RM2235, Leica, Germany). Then, an intricate process involving deparaffinization, rehydration, and H&E staining was undertaken to prepare these samples for further analysis under the microscope (EVOS5000, Invitrogen, USA).

### 4.22 Immunofluorescence Staining of Tissue Section

Wound tissue samples from the IC, ASC, Gel, and Gel‐AS groups on D2 and D7 were collected and embedded in paraffin. The embedded samples were then sectioned into 4 µm thick slices using a paraffin slicing machine (RM2235, Leica, Germany), and these sections were affixed to polylysine‐treated slides. After overnight baking, the sections were dewaxed and rehydrated. For antigen repair, the samples were placed into a microwave‐safe repair cassette filled with citric acid antigen repair buffer (pH 6.0) and then blocked with BSA. The primary antibodies of F4/80 (1:200), CD86 (1:200), CD206 (1:200), TNF‐α (1:200) or FTH1 (1:200) were applied to cover the sections. Furthermore, some sections were incubated with FPN1 antibody (1:200) overnight at 4°C. The subsequent steps were conducted according to the immunofluorescence methodology previously outlined in above. The resulting images were captured using a microscope (EVOS5000, Invitrogen, USA).

### 4.23 Antimicrobial Activity Test

*S. aureus* (BNCC 186335) was revived by incubating its freezing solutions with Luria−Bertani broth medium (LB, OXOID, UK) on the shaker (37°C, 24 h). Then, the revived suspensions were re‐cultured in another LB broth medium at 37°C for 16−18 h up to their logarithmic phase (1 × 10^8^ CFU mL^−1^). Next, 0.1 mL of PBS or Gel‐AS‐IS were added to the suspensions of *S. aureus* and incubated for another 24 h. After incubation, the mixture (0.2 mL) was cultured on nutrient agar and incubated at 37°C for 24 h. The colonies of *S. aureus* treated with PBS and Gel‐AS‐IS were recorded by taking photographs.

### 4.24 Statistical Analysis

All experimental data analysis were performed using GraphPad Prism 8.4.3 (GraphPad Software). The data is presented as mean ± standard deviation (mean ± SD). The number of independent trials has been specified in the corresponding figure legends. The Student's *t*‐test was employed to analyze the differences between two groups. One‐way ANOVA analysis was used to assess variances among multiple groups. Specific statistical information for each experiment can be found in the corresponding figure legends. Differences with *p‐*values less than 0.05 were considered significant.

## Author Contributions

Shengnan Cui, Xi Liu, Cuiping Zhang, and Xiaobing Fu conceived the idea, designed the experiments, supervised, and funded the research. Shengnan Cui, Yong Liu, and Sheng Meng conducted most of the experiments. Weicheng Hu, Liqian Ma, and Shengqiu Chen performed the Western blotting assay and analysis. Qilin Huang and Ziqiang Chu assisted with in vitro and in vivo experiments. Weicheng Zhong analyzed results. Xi Liu wrote the manuscript. Xi Liu, Shengnan Cui, Sheng Meng, Yufeng Jiang, and Cuiping Zhang revised the figures and the manuscript.

## Conflicts of Interest

The authors declare no conflicts of interests.

## Ethics Statement

All the animal experiments were performed in Beijing View Solid Biotechnology Co., Ltd, (Beijing, China) and approved by the Ethics Committee of Beijing View solid Biotechnology Co. Ltd, (Approval number: vs2126A 00724).

## Supporting information




**Supporting File 1**: exp270092‐sup‐0001‐SuppMat.docx.

## Data Availability

All data supporting the findings of this study are available from the corresponding authors upon reasonable request.

## References

[1] A. Zacharias , T. A. Schwann , C. J. Riordan , S. J. Durham , A. S. Shah , and R. H. Habib , “Obesity and Risk of New‐Onset Atrial Fibrillation After Cardiac Surgery,” Circulation 112 (2005): 3247–3255, 10.1161/CIRCULATIONAHA.105.553743. 16286585

[2] X. Liu , Q. Wei , Z. Q. Sun , et al., “Small Extracellular Vesicles: Yields, Functionalization and Applications in Diabetic Wound Management,” Interdisciplinary Medicine 1, no. 4 (2023): 0230019, 10.1002/INMD.20230019.

[3] T. F. Ma , X. Y. Zhai , M. D. Jin , et al., “Multifunctional Wound Dressing for Highly Efficient Treatment of Chronic Diabetic Wounds,” View 3, no. 6 (2022): 20220045, 10.1002/VIW.20220045.

[4] Q. Li , W. Hu , Q. Huang , et al., “MiR146a‐Loaded Engineered Exosomes Released From Silk Fibroin Patch Promote Diabetic Wound Healing by Targeting IRAK1,” Signal Transduction and Targeted Therapy 8 (2023): 62, 10.1038/s41392-022-01263-w. 36775818 PMC9922687

[5] Z. Chu , Q. Huang , K. Ma , et al., “Novel Neutrophil Extracellular Trap‐Related Mechanisms in Diabetic Wounds Inspire a Promising Treatment Strategy With Hypoxia‐Challenged Small Extracellular Vesicles,” Bioactive Materials 27 (2023): 257–270. 37122894 10.1016/j.bioactmat.2023.04.007 PMC10133407

[6] Y. Wang , Z. Cao , Q. Wei , et al., “VH298‐Loaded Extracellular Vesicles Released From Gelatin Methacryloyl Hydrogel Facilitate Diabetic Wound Healing by HIF‐1α‐Mediated Enhancement of Angiogenesis,” Acta Biomaterialia 147 (2022): 342–355, 10.1016/j.actbio.2022.05.018. 35580827

[7] X. L. Qi , E. Cai , Y. J. Xiang , et al., “An Immunomodulatory Hydrogel by Hyperthermia‐Assisted Self‐Cascade Glucose Depletion and ROS Scavenging for Diabetic Foot Ulcer Wound Therapeutics,” Advanced Materials 35, no. 48 (2023): 2306632. 10.1002/adma.202306632 37803944

[8] S. J. Lee , C. K. Lee , S. Kang , et al., “Angiopoietin‐2 Exacerbates Cardiac Hypoxia and Inflammation After Myocardial Infarction,” Journal of Clinical Investigation 128 (2018): 5018–5033, 10.1172/JCI99659. 30295643 PMC6205384

[9] L. C. Chan , M. Rossetti , L. S. Miller , et al., “Protective immunity in recurrent Staphylococcus aureus infection reflects localized immune signatures and macrophage‐conferred memory,” PNAS 115 (2018): E11111–E11119. 30297395 10.1073/pnas.1808353115 PMC6255181

[10] Y. Guo , C. Lin , P. Xu , et al., “AGEs Induced Autophagy Impairs Cutaneous Wound Healing via Stimulating Macrophage Polarization to M1 in Diabetes,” Scientific Reports 6 (2016): 36416, 10.1038/srep36416. 27805071 PMC5090347

[11] S. Devaraj , S. K. Venugopal , U. Singh , and I. Jialal , “Hyperglycemia Induces Monocytic Release of Interleukin‐6 via Induction of Protein Kinase C‐α and ‐β,” Diabetes 54 (2005): 85–91, 10.2337/diabetes.54.1.85. 15616014

[12] S. Pavlou , J. Lindsay , R. Ingram , H. Xu , and M. Chen , “Sustained High Glucose Exposure Sensitizes Macrophage Responses to Cytokine Stimuli but Reduces Their Phagocytic Activity,” BMC Immunology 19 (2018): 24, 10.1186/s12865-018-0261-0. 29996768 PMC6042333

[13] E. Cai , X. L. Qi , Y. Z. Shi , et al., “Immunomodulatory Melanin@Pt Nanoparticle‐Reinforced Adhesive Hydrogels for Healing Diabetic Oral Ulcers,” Chemical Engineering Journal 488 (2024): 150372, 10.1016/j.cej.2024.150372.

[14] X. Qi , E. Cai , Y. Xiang , et al., “An Immunomodulatory Hydrogel by Hyperthermia‐Assisted Self‐Cascade Glucose Depletion and ROS Scavenging for Diabetic Foot Ulcer Wound Therapeutics,” Advanced Materials 35, no. 48 (2023): e2306632, 10.1002/adma.202306632. 37803944

[15] Y. J. Xiang , X. L. Qi , E. Cai , et al., “Highly Efficient Bacteria‐Infected Diabetic Wound Healing Employing a Melanin‐Reinforced Biopolymer Hydrogel,” Chemical Engineering Journal 460 (2023): 141852–141852, 10.1016/j.cej.2023.141852.

[16] J. He , W. Zhang , Y. Cui , L. Cheng , X. L. Chen , and X. Wang , “Multifunctional Cu 2 Se/F127 Hydrogel With SOD‐Like Enzyme Activity for Efficient Wound Healing,” Advanced Healthcare Materials 13, no. 16 (2024): e2303599, 10.1002/adhm.202303599. 38331398

[17] Y. Cui , W. Zhang , J. Shan , et al., “Copper Nanodots‐Based Hybrid Hydrogels With Multiple Enzyme Activities for Acute and Infected Wound Repair,” Advanced Healthcare Materials 13, no. 8 (2024): e2302566, 10.1002/adhm.202302566. 37931140

[18] H. Tian , J. Yan , W. Zhang , et al., “Cu‐GA‐Coordination Polymer Nanozymes With Triple Enzymatic Activity for Wound Disinfection and Accelerated Wound Healing,” Acta Biomaterialia 167 (2023): 449–462, 10.1016/j.actbio.2023.05.048. 37270076

[19] J. Han , X. Jin , C. Zhang , et al., “Metal Natural Product Complex Ru‐procyanidins With Quadruple Enzymatic Activity Combat Infections From Drug‐Resistant Bacteria,” Acta Pharmaceutica Sinica B 14, no. 5 (2024): 2298–2316, 10.1016/j.apsb.2023.12.017. 38799629 PMC11121202

[20] X. Jin , W. Zhang , J. Shan , et al., “Thermosensitive Hydrogel Loaded With Nickel–Copper Bimetallic Hollow Nanospheres With SOD and CAT Enzymatic‐Like Activity Promotes Acute Wound Healing,” ACS Applied Materials and Interfaces 14, no. 45 (2022): 50677–50691, 10.1021/acsami.2c17242. 36326126

[21] A. P. Veith , K. Henderson , A. Spencer , A. D. Sligar , and A. B. Baker , “Therapeutic Strategies for Enhancing Angiogenesis in Wound Healing,” Advanced Drug Delivery Reviews 146 (2019): 97–125, 10.1016/j.addr.2018.09.010. 30267742 PMC6435442

[22] J. Jin , Z. Zhang , J. Chen , Y. Liu , Q. Chen , and Q. Wang , “Jixuepaidu Tang‐1 Inhibits Epithelial‐Mesenchymal Transition and Alleviates Renal Damage in DN Mice Through Suppressing Long Non‐Coding RNA LOC498759,” Cell Cycle 18 (2019): 3125–3136, 10.1080/15384101.2019.1669986. 31564202 PMC6816411

[23] C. Puglia , M. R. Lauro , G. G. Tirendi , et al., “Modern Drug Delivery Strategies Applied to Natural Active Compounds,” Expert Opinion on Drug Delivery 14 (2017): 755–768, 10.1080/17425247.2017.1234452. 27606793

[24] Y. Y. Huang , C. W. Lin , N. C. Cheng , et al., “Effect of a Novel Macrophage‐Regulating Drug on Wound Healing in Patients with Diabetic Foot Ulcers,” JAMA Network Open 4, no. 9 (2021): e2122607, 10.1001/jamanetworkopen.2021.22607. 34477854 PMC8417758

[25] D. Paolino , D. Cosco , F. Cilurzo , et al., “Improved In Vitro and In Vivo Collagen Biosynthesis by Asiaticoside‐Loaded Ultradeformable Vesicles,” Journal Control Release 162 (2012): 143–151, 10.1016/j.jconrel.2012.05.050. 22698941

[26] H. Li , Q. Peng , Y. Guo , X. Wang , and L. Zhang , “Preparation and In Vitro and In Vivo Study of Asiaticoside‐Loaded Nanoemulsions and Nanoemulsions‐Based Gels for Transdermal Delivery,” The International Journal of Nanomedicine 15 (2020): 3123–3136, 10.2147/IJN.S241923. 32440114 PMC7210032

[27] J. Wan , X. Gong , R. Jiang , Z. Zhang , and L. Zhang , “Antipyretic and Anti‐Inflammatory Effects of Asiaticoside in Lipopolysaccharide‐Treated Rat Through Up‐Regulation of Heme Oxygenase‐1,” Phytotherapy Research 27 (2013): 1136–1142, 10.1002/ptr.4838. 22972613

[28] J. Qiu , L. Yu , X. Zhang , et al., “Asiaticoside Attenuates Lipopolysaccharide‐Induced Acute Lung Injury via Down‐Regulation of NF‐κB Signaling Pathway,” International Immunopharmacology 26 (2015): 181–187, 10.1016/j.intimp.2015.03.022. 25835778

[29] D. K. Li and G. H. Wang , “Asiaticoside Reverses M2 Phenotype Macrophage Polarization‐Evoked Osteosarcoma Cell Malignant Behaviour by TRAF6/NF‐κB Inhibition,” Pharmaceutical Biology 60 (2022): 1635–1645, 10.1080/13880209.2022.2109688. 35989576 PMC9415541

[30] S. Cui , X. Liu , Y. Liu , et al., “Autophagosomes Defeat Ferroptosis by Decreasing Generation and Increasing Discharge of Free Fe^2+^ in Skin Repair Cells to Accelerate Diabetic Wound Healing,” Advanced Science 10 (2023): e2300414, 10.1002/advs.202300414. 37387572 PMC10477857

[31] R. Kim , A. Hashimoto , N. Markosyan , et al., “Ferroptosis of Tumour Neutrophils Causes Immune Suppression in Cancer,” Nature 612 (2022): 338–346, 10.1038/s41586-022-05443-0. 36385526 PMC9875862

[32] Y. Wu , H. Chen , N. Xuan , et al., “Induction of Ferroptosis‐Like Cell Death of Eosinophils Exerts Synergistic Effects With Glucocorticoids in Allergic Airway Inflammation,” Thorax 75 (2020): 918–927, 10.1136/thoraxjnl-2020-214764. 32759385

[33] Y. Cui , Z. Zhang , X. Zhou , et al., “Microglia and Macrophage Exhibit Attenuated Inflammatory Response and Ferroptosis Resistance After RSL3 Stimulation via Increasing Nrf2 Expression,” Journal Neuroinflammation 18 (2021): 249, 10.1186/s12974-021-02231-x. PMC8557003 34717678

[34] Y. Yang , Y. Wang , L. Guo , W. Gao , T. L. Tang , and M. Yan , “Interaction Between Macrophages and Ferroptosis,” Cell Death & Disease 13 (2022): 355, 10.1038/s41419-022-04775-z. 35429990 PMC9013379

[35] A. A. Kapralov , Q. Yang , H. H. Dar , et al., “Redox Lipid Reprogramming Commands Susceptibility of Macrophages and Microglia to Ferroptotic Death,” Nature Chemical Biology 16 (2020): 278–290, 10.1038/s41589-019-0462-8. 32080625 PMC7233108

[36] F. Yu , Q. Zhang , H. Liu , et al., “Dynamic O‐GlcNAcylation Coordinates Ferritinophagy and Mitophagy to Activate Ferroptosis,” Cell Discovery 8 (2022): 40, 10.1038/s41421-022-00390-6. 35504898 PMC9065108

[37] F. Wang , H. Lv , B. Zhao , et al., “Iron and Leukemia: New Insights for Future Treatments,” Journal of Experimental & Clinical Cancer Research 38 (2019): 406, 10.1186/s13046-019-1397-3. 31519186 PMC6743129

[38] P. Qin , J. Tang , D. Sun , et al., “Zn^2+^ Cross‐Linked Alginate Carrying Hollow Silica Nanoparticles Loaded With RL‐QN15 Peptides Provides Promising Treatment for Chronic Skin Wounds,” ACS Applied Materials and Interfaces 14, no. 26 (2022): 29491–29505, 10.1021/acsami.2c03583. 35731847

[39] C. B. Ibberson , A. Stacy , D. Fleming , et al., “Co‐Infecting Microorganisms Dramatically Alter Pathogen Gene Essentiality During Polymicrobial Infection,” Nature Microbiology 2 (2017): 17079, 10.1038/nmicrobiol.2017.79. 30. PMC5774221 28555625

[40] C. Chen , L. Chen , C. Mao , et al., “Natural Extracts for Antibacterial Applications,” Small 20, no. 9 (2024): e2306553, 10.1002/smll.202306553. 37847896

[41] G. Courties , T. Heidt , M. Sebas , et al., “In Vivo Silencing of the Transcription Factor IRF5 Reprograms the Macrophage Phenotype and Improves Infarct Healing,” Journal of the American College of Cardiology 63 (2014): 1556–1566, 10.1016/j.jacc.2013.11.023. 24361318 PMC3992176

[42] D. G. Armstrong , T. W. Tan , A. J. M. Boulton , and S. A. Bus , “Diabetic Foot Ulcers,” JAMA 330 (2023): 62, 10.1001/jama.2023.10578. 37395769 PMC10723802

[43] J. T. Paige , M. Kremer , J. Landry , et al., “Modulation of Inflammation in Wounds of Diabetic Patients Treated With Porcine Urinary Bladder Matrix,” Regenerative Medicine 14 (2019): 269–277, 10.2217/rme-2019-0009. 31020913 PMC6886567

[44] K. D. Mangum and K. A. Gallagher , “Obesity Confers Macrophage Memory,” Science 379 (2023): 28–29, 10.1126/science.adf6582. 36603093

[45] M. Morey , P. O'Gaora , A. Pandit , and C. Helary , “Hyperglycemia Acts in Synergy With Hypoxia to Maintain the Pro‐Inflammatory Phenotype of Macrophages,” PLoS ONE 14 (2019): e0220577, 10.1371/journal.pone.0220577. 31415598 PMC6695165

[46] S. Basu Mallik , B. S. Jayashree , and R. R. Shenoy , “Epigenetic Modulation of Macrophage Polarization‐Perspectives in Diabetic Wounds,” Journal of Diabetes and Its Complications 32 (2018): 524–530, 10.1016/j.jdiacomp.2018.01.015. 29530315

[47] F. Bohm , U. A. Kohler , T. Speicher , and S. Werner , “Regulation of Liver Regeneration by Growth Factors and Cytokines,” EMBO Molecular Medicine 2 (2010): 294–305, 10.1002/emmm.201000085. 20652897 PMC3377328

[48] S. Lakoh , L. Yi , S. Sevalie , et al., “Incidence and Risk Factors of Surgical Site Infections and Related Antibiotic Resistance in Freetown, Sierra Leone: A Prospective Cohort Study,” Antimicrobial Resistance and Infection Control 11 (2022): 39, 10.1186/s13756-022-01078-y. 35189952 PMC8862228

[49] N. E. Paul , C. Skazik , M. Harwardt , et al., “Topographical Control of Human Macrophages by a Regularly Microstructured Polyvinylidene Fluoride Surface,” Biomaterials 29 (2008): 4056–4064, 10.1016/j.biomaterials.2008.07.010. 18667233

[50] C. Li , Y. Xiong , Z. Fu , et al., “The Direct Binding of Bioactive Peptide Andersonin‐W1 to TLR4 Expedites the Healing of Diabetic Skin Wounds,” Cellular & Molecular Biology Letters 29 (2024): 24, 10.1186/s11658-024-00542-4. 38317065 PMC10845795

[51] C. Li , Z. Fu , T. Jin , et al., “A Frog Peptide Provides New Strategies for the Intervention Against Skin Wound Healing,” Cellular & Molecular Biology Letters 28 (2023): 61, 10.1186/s11658-023-00468-3. 37501100 PMC10375744

[52] F. X. Zhu , G. G. Nie , and C. S. Liu , “Engineered Biomaterials in Stem Cell‐Based Regenerative Medicine,” Life Medicine 2, no. 4 (2023): 1–22, 10.1093/lifemedi/lnad027. 20. PMC11749850 39872549

[53] C. Li , Z. Fu , T. Jin , et al., “A Frog Peptide Provides New Strategies for the Intervention Against Skin Wound Healing,” Cellular & Molecular Biology Letters 28, no. 1 (2023): 61, 10.1186/s11658-023-00468-3. 37501100 PMC10375744

[54] C. Li , Y. Xiong , Z. Fu , et al., “The Direct Binding of Bioactive Peptide Andersonin‐W1 to TLR4 Expedites the Healing of Diabetic Skin Wounds,” Cellular & Molecular Biology Letters 29, no. 1 (2024): 24, 10.1186/s11658-024-00542-4. 5. 38317065 PMC10845795

[55] Y. Wang , B. Tang , J. Zhu , et al., “Emerging Mechanisms and Targeted Therapy of Ferroptosis in Neurological Diseases and Neuro‐Oncology,” International Journal of Biological Sciences 18 (2022): 4260–4274, 10.7150/ijbs.72251. 35844784 PMC9274504

[56] S. Xu , J. Min , and F. Wang , “Ferroptosis: An Emerging Player in Immune Cells,” Science Bulletin 66 (2021): 2257–2260, 10.1016/j.scib.2021.02.026. 36654451

[57] Y. S. Sohn , A. M. Mitterstiller , W. Breuer , G. Weiss , and Z. I. Cabantchik , “Rescuing Iron‐Overloaded Macrophages by Conservative Relocation of the Accumulated Metal,” British Journal of Pharmacology 164 (2011): 406–418, 10.1111/j.1476-5381.2010.01120.x. 21091647 PMC3188895

[58] Y. Yuan , Y. Chen , T. Peng , et al., “Mitochondrial ROS‐induced Lysosomal Dysfunction Impairs Autophagic Flux and Contributes to M1 Macrophage Polarization in a Diabetic Condition,” Clinical Science 133 (2019): 1759–1777, 10.1042/CS20190672. 31383716

[59] B. Levine and G. Kroemer , “Biological Functions of Autophagy Genes: A Disease Perspective,” Cell 176 (2019): 11–42, 10.1016/j.cell.2018.09.048. 30633901 PMC6347410

[60] J. Füllgrabe , D. J. Klionsky , and B. Joseph , “The Return of the Nucleus: Transcriptional and Epigenetic Control of Autophagy,” Nature Reviews Molecular Cell Biology 15 (2014): 65–74, 10.1038/nrm3716. 24326622

[61] F. Zheng , R. Li , Q. He , et al., “The Electrostimulation and Scar Inhibition Effect of Chitosan/Oxidized Hydroxyethyl Cellulose/Reduced Graphene Oxide/Asiaticoside Liposome Based Hydrogel on Peripheral Nerve Regeneration In Vitro,” Materials Science & Engineering C Materials for Biological Applications 109 (2020): 110560, 10.1016/j.msec.2019.110560. 32228996

[62] H. Bunzen and D. Jirák , “Recent Advances in Metal–Organic Frameworks for Applications in Magnetic Resonance Imaging,” ACS Applied Materials & Interfaces 14, no. 45 (2022): 50445–50462, 10.1021/acsami.2c10272. 36239348 PMC10749454

[63] T. Wu , C. Cui , C. Fan , Z. Xu , Y. Liu , and W. Liu , “Tea Eggs‐Inspired High‐Strength Natural Polymer Hydrogels,” Bioactive Materials 6 (2021): 2820. 33718664 10.1016/j.bioactmat.2021.02.009 PMC7903155

[64] M. Xie , Y. Shi , C. Zhang , et al., “In Situ 3D Bioprinting With Bioconcrete Bioink,” Nature Communications 13 (2022): 3597, 10.1038/s41467-022-30997-y. PMC9225998 35739106

[65] Z. Zhuang , Y. Zhang , S. Sun , et al., “Control of Matrix Stiffness Using Methacrylate–Gelatin Hydrogels for a Macrophage‐Mediated Inflammatory Response,” ACS Biomaterials Science and Engineering 6, no. 5 (2020): 3091–3102, 10.1021/acsbiomaterials.0c00295. 33463297

[66] S. Butenko , R. R. Nagalla , C. F. Guerrero‐Juarez , et al., “Hydrogel Crosslinking Modulates Macrophages, Fibroblasts, and Their Communication, During Wound Healing,” Nature Communications 15, no. 1 (2024): 6820, 10.1038/s41467-024-50072-y. PMC11315930 39122702

