# Supplementary material for: Activating Ferroptosis of M1 Macrophages: A Novel Mechanism of Asiaticoside Encapsuled in GelMA for Anti‐Inflammation in Diabetic Wounds
Source: Exploration (Beijing). 2025 Oct 31;5(6):20240062. doi: 10.1002/EXP.20240062 (PMC12752607; doi:10.1002/EXP.20240062)
Supplement: Supplementary file 1 — Supporting File 1: exp270092‐sup‐0001‐SuppMat.docx. [file EXP2-5-20240062-s001.docx]

**Activating ferroptosis of M1 macrophages: a novel mechanism of asiaticoside encapsuled in GelMA for anti-inflammation in diabetic wounds**

Shengnan Cui^1,2†^, Sheng Meng^1†^, Yong Liu^3†^, Shengqiu Chen^4^, Wenzhi Hu^1,5,6^, Qilin Huang^1^, Ziqiang Chu^1^, Weicheng Zhong^1^, Liqian Ma^1,5,6^, Zhe Li^7^, Yufeng Jiang^8*^, Xi Liu^1,5,6*^, Xiaobing Fu^1,5,6*^, Cuiping Zhang^1,5,6*^

^1^ Medical Innovation Research Department, PLA General Hospital and PLA Medical College, Beijing, 100048, China.

^2^ Department of Dermatology, The Second Affiliated Hospital, Shaanxi University of Chinese Medicine, Xianyang, Shaanxi, 712046, China.

^3^ Department of Dermatology, The Second Affiliated Hospital of Xi’ an Jiaotong University, Xi’an, Shaanxi, 710004, China.

^4^ Innovation Research Center for Diabetic Foot, West China Hospital, Sichuan University, Chengdu, 610041, China.

^5^ PLA Key Laboratory of Tissue Repair and Regenerative Medicine and Beijing Key Research Laboratory of Skin Injury, Repair and Regeneration, Beijing, 100048, China.

^6^ Research Unit of Trauma Care, Tissue Repair and Regeneration, Chinese Academy of Medical Sciences, Beijing, 100048, China.

^7^ Burns Unit, Concord Hospital, University of Sydney Medical School, Australia.

^8^ Department of Tissue Regeneration and Wound Repair, PLA General Hospital, Beijing, 100048, China.

† These authors contributed equally: Shengnan Cui, Sheng Meng, and Yong Liu.

* Corresponding authors:

Yufeng Jiang

Department of Tissue Regeneration and Wound Repair, PLA General Hospital, Beijing, 100048, China.

Email: fisherman306@qq.com;

Xi Liu, Xiaobing Fu, and Cuiping Zhang

Medical Innovation Research Department, PLA General Hospital and PLA Medical College, Beijing, 100048, China.

Email: [liuxipla@163.com](mailto:liuxipla@163.com); [fuxiaobing@vip.sina.com](mailto:fuxiaobing@vip.sina.com); zcp666666@sohu.com.


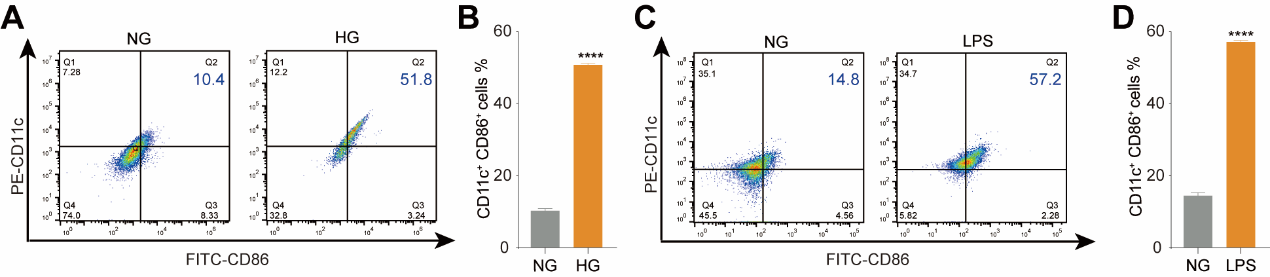


**Figure S1.** The proportion of CD11c^+^ CD86^+^ cells in HG- and LPS-M1 macrophages. (A) FCM images showing the proportion of CD11c^+^CD86^+^ cells in NG macrophages and HG-M1 macrophages. (B) Quantitative analysis of the proportion of CD11c^+^ CD86^+^ cells in (A) (Mean ± SD; Student’s *t*-test, n = 3). (C) FCM images depicting the proportion of CD11c^+^ CD86^+^ cells in NG macrophages and LPS-M1 macrophages. (D) Quantitative analysis of the proportion of CD11c^+^ CD86^+^ cells in (C) (Mean ± SD; Student’s *t*-test, n = 3). Statistically significant differences between groups are indicated as follows: *****p* < 0.0001.


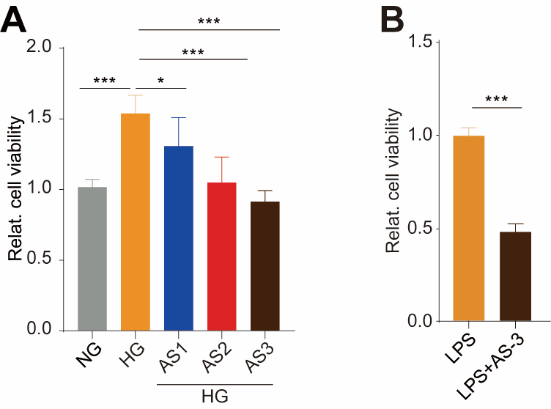


**Figure S2.** The role of AS in determining the cell viability of (A) HG-M1 macrophages (Mean ± SD; one-way ANOVA, n = 6) and (B) LPS-M1 macrophages (Mean ± SD; Student’s *t*-test, n = 6). Statistically significant differences between groups are indicated as follows: **p* < 0.05, ****p* < 0.001.

**
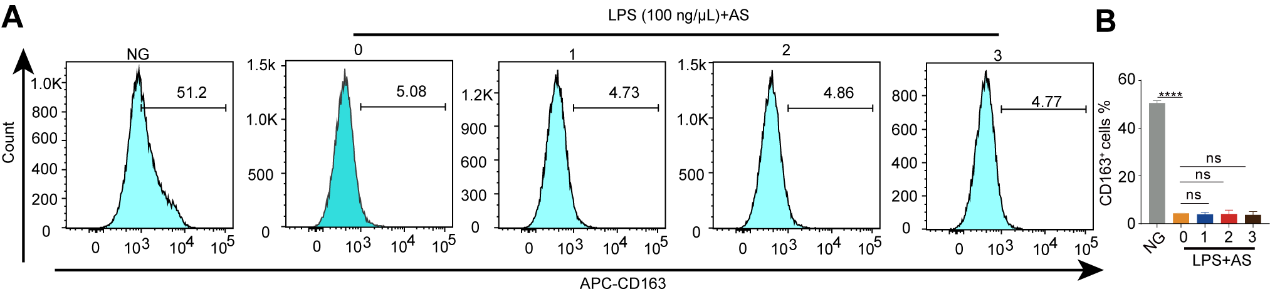
**

**Figure S3.** The effect of AS on the proportion of CD163^+^ macrophages in LPS-M1 macrophages. (A) FCM images showing the proportion of CD163^+^ cells in NG macrophages and AS-treated LPS-M1 macrophages. (B) Quantitative analysis of CD163^+^ cells in (A) (Mean ± SD; one-way ANOVA, n = 3). Statistically significant differences between groups are indicated as follows: ns, not significant, ****p* < 0.001.

**
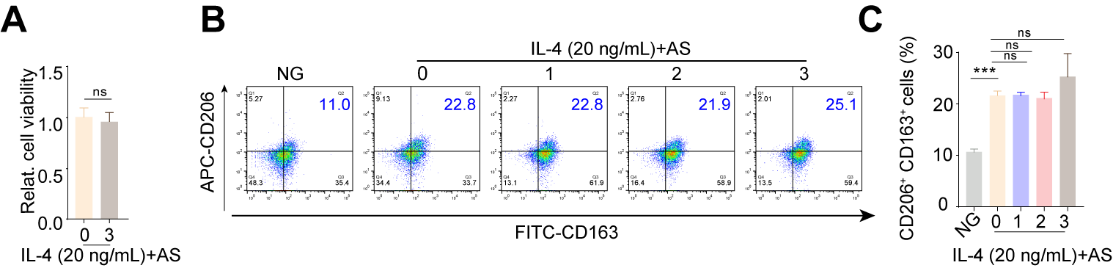
**

**Figure S4.** AS didn’t affect the viability and the proportion of CD163^+^CD206^+^ cells of M2 macrophages. (A) The cell viability of AS3-treated M2 macrophages (Mean ± SD; Student’s *t*-test, n = 3). (B) FCM images showing the relative ratio of CD163^+^CD206^+^ cells in NG macrophages, and IL-4-pretreated M2 macrophages with the treatment of AS1, AS2, and AS3. (C) Quantitative analysis of CD163^+^CD206^+^ cells in (B) (Mean ± SD; one-way ANOVA, n = 3). Statistically significant differences between groups are indicated as follows: ns, not significant, ****p* < 0.001.

**
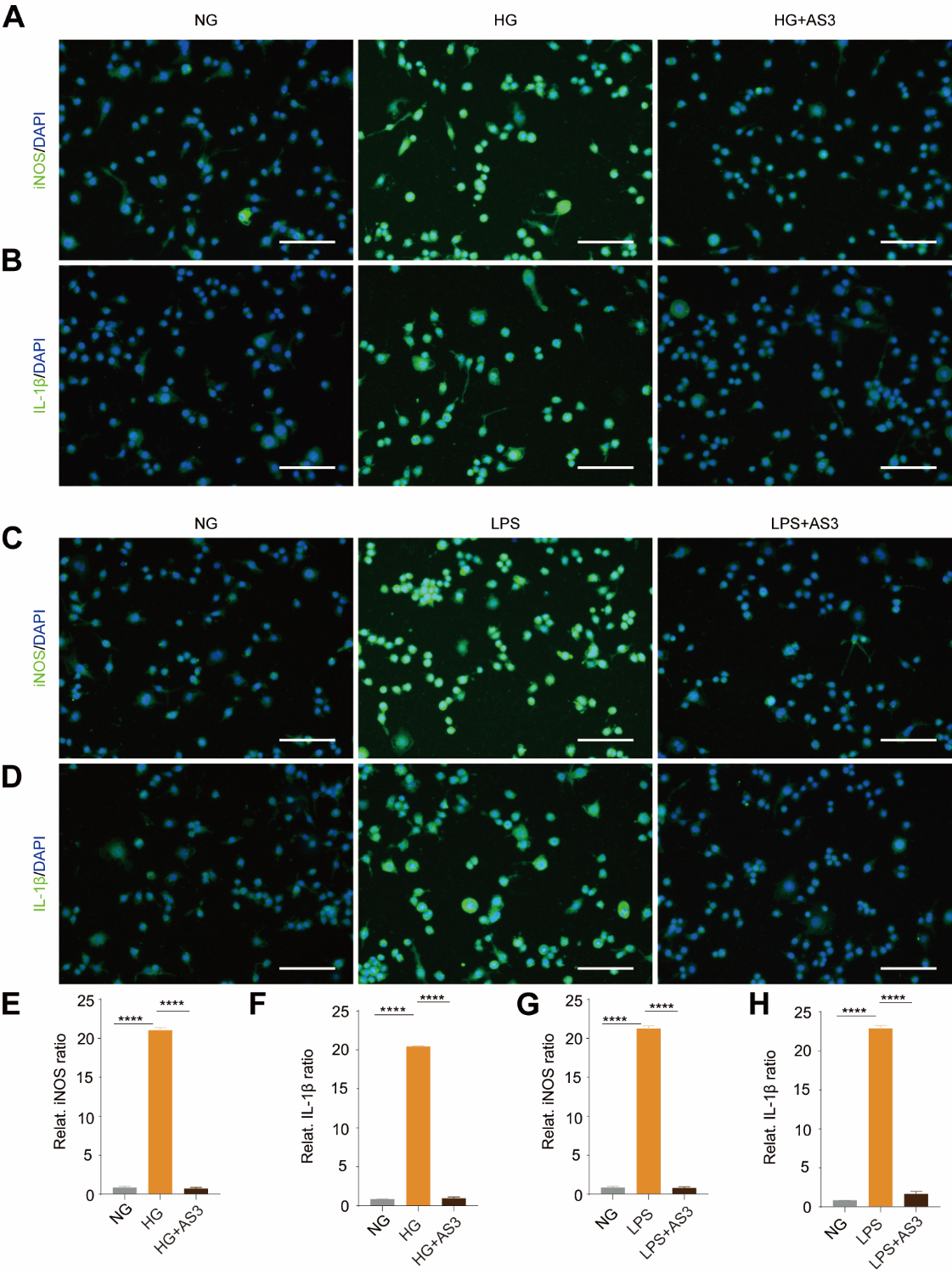
**

**Figure S5.** AS decreased the secretion of iNOS and IL-1β proteins in HG-M1 and LPS-M1 macrophages. (A) Fluorescent images and (E) quantitative analysis reflecting iNOS expression in HG-M1 macrophages (Mean ± SD; one-way ANOVA, n = 3). Scale bar, 100 μm. (B) Fluorescent images and (F) quantitative analysis reflecting IL-1β expression in HG-M1 macrophages (Mean ± SD; one-way ANOVA, n = 3). Scale bar, 100 μm. (C) Fluorescent images and (G) quantitative analysis reflecting iNOS expression in LPS-M1 macrophages (Mean ± SD; one-way ANOVA, n = 3). Scale bar, 100 μm. (D) Fluorescent images and (H) quantitative analysis reflecting IL-1β expression in LPS-M1 macrophages (Mean ± SD; one-way ANOVA, n = 3). Cell nucleus was dyed with DAPI (blue fluorescence). Scale bar, 100 μm. Statistically significant differences between groups are indicated as follows: *****p* < 0.0001.


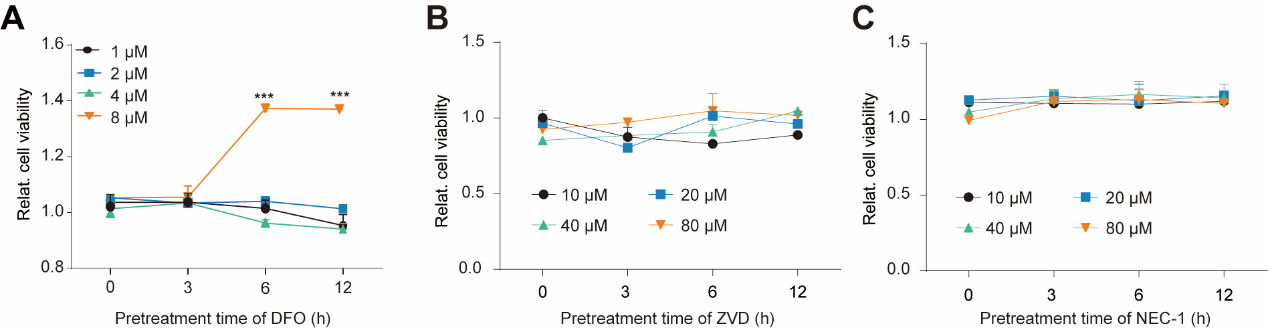


**Figure S6.** CCK-8 assay showing the effect of DFO (A), ZVD (B) and NEC-1 (C) pretreatment on the cell viability of HG-M1 macrophages with the post-treatment of AS3 (Mean ± SD; one-way ANOVA, n = 6). Statistically significant differences between groups are indicated as follows: ****p* < 0.001.


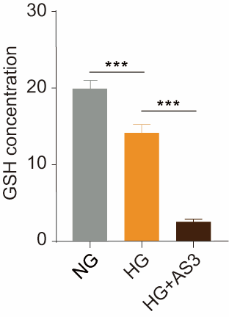


**Figure S7.** The concentration of GSH in NG macrophages, HG-M1 macrophages, and AS3-treated HG-M1 macrophages (Mean ± SD; one-way ANOVA, n = 3). Statistically significant differences between groups are indicated as follows: ****p* < 0.001.


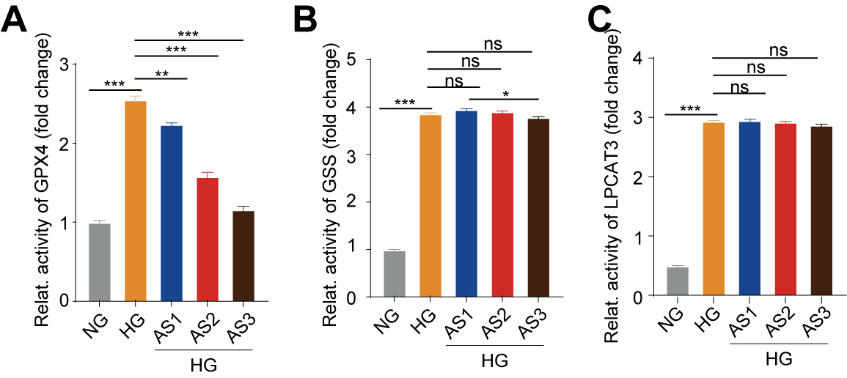


**Figure S8.** The effect of AS on the catalytic activities of (A) GPX4, (B) GSS, and (C) LPCAT3 in HG-M1 macrophages. (Mean ± SD; one-way ANOVA, n = 3). Statistically significant differences between groups are indicated as follows: ns, not significant, **p* < 0.05, ***p* < 0.01, ****p* < 0.001.


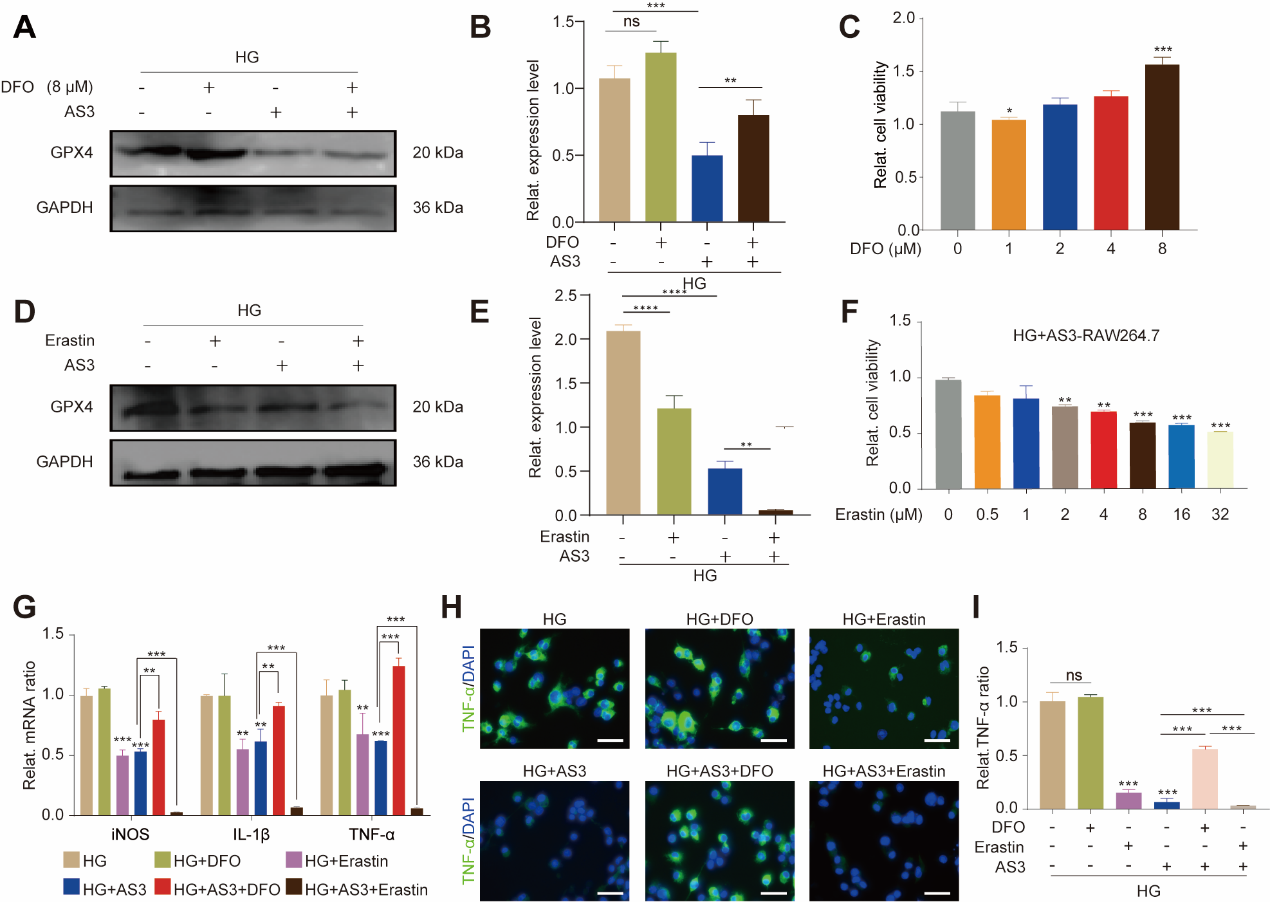


**Figure S9.** The role of ferroptosis in mediating the immunoregulatory effect of AS on HG-M1 macrophages. (A) Western blotting images and (B) quantitative band intensities showing the expression level of GPX4 protein in HG-M1 macrophages with different treatments (Mean ± SD; one-way ANOVA, n = 3). (C) CCK-8 assay showing the effect of DFO-pretreatment on the cell viability of HG-M1 macrophages with the posttreatment of AS3 (Mean ± SD; one-way ANOVA, n = 6). (D) Western blotting images and (E) quantitative band intensities displaying the expression level of GPX4 protein in HG-M1 macrophages with different treatments (Mean ± SD; one-way ANOVA, n = 3). (F) CCK-8 assay reflecting the effect of Erastin-pretreatment for 6 h on the cell viability of HG-M1 macrophages with the posttreatment of AS3 (Mean ± SD; one-way ANOVA, n = 6). (G) qRT-PCR results exhibiting the gene expression levels of iNOS, IL-1β, and TNF-α in HG-M1 macrophages under various conditions (Mean ± SD; one-way ANOVA, n = 3). (H) Fluorescent images and (I) quantitative statistics reflecting the expression of TNF-α protein inside HG-M1 macrophages with different treatments (Mean ± SD; one-way ANOVA, n = 3). Scale bar, 100 μm. Cell nucleus was dyed with Hoechst33342 (blue fluorescence). Statistically significant differences between groups are indicated as follows: ns, not significant, **p* < 0.05, ***p* < 0.01, ****p* < 0.001,*****p* < 0.0001.


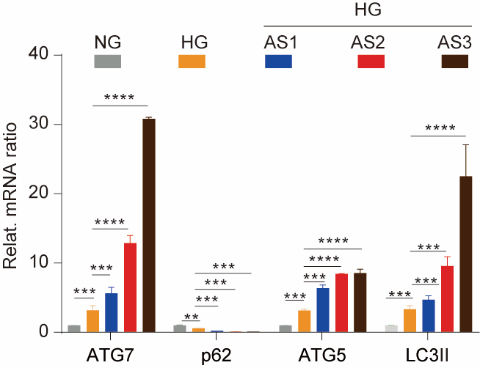


**Figure S10.** qRT-PCR results reflecting the expression levels of ATG7, p62, ATG5, and LC3II genes inside NG macrophages, HG-M1 macrophages, and AS-treated HG-M1 macrophages **(**Mean ± SD; one-way ANOVA, n = 3**)**. Statistically significant differences between groups are indicated as follows: ***p* < 0.01, ****p* < 0.001, *****p* < 0.0001.


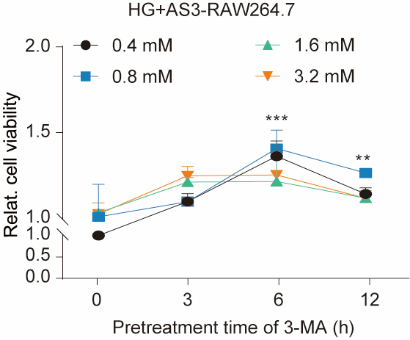


**Figure S11.** CCK-8 assay evaluating the effect of 3-MA-pretreatment on cell viability of HG-M1 macrophages with the post-treatment of AS3 (Mean ± SD; one-way ANOVA, n = 6). Statistically significant differences between groups are indicated as follows: ***p* < 0.01, ****p* < 0.001.


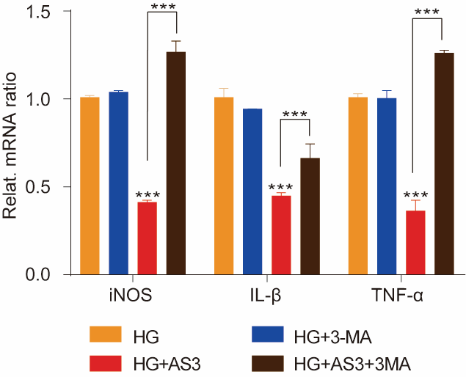


**Figure S12.** qRT-PCR results demonstrating the gene expression levels of iNOS, IL-1β, and TNF-α inside 3-MA-pretreated HG-M1 macrophages with or without the post-treatment of AS3 **(**Mean ± SD; one-way ANOVA, n = 3). Statistically significant differences between groups are indicated as follows: ****p* < 0.001.


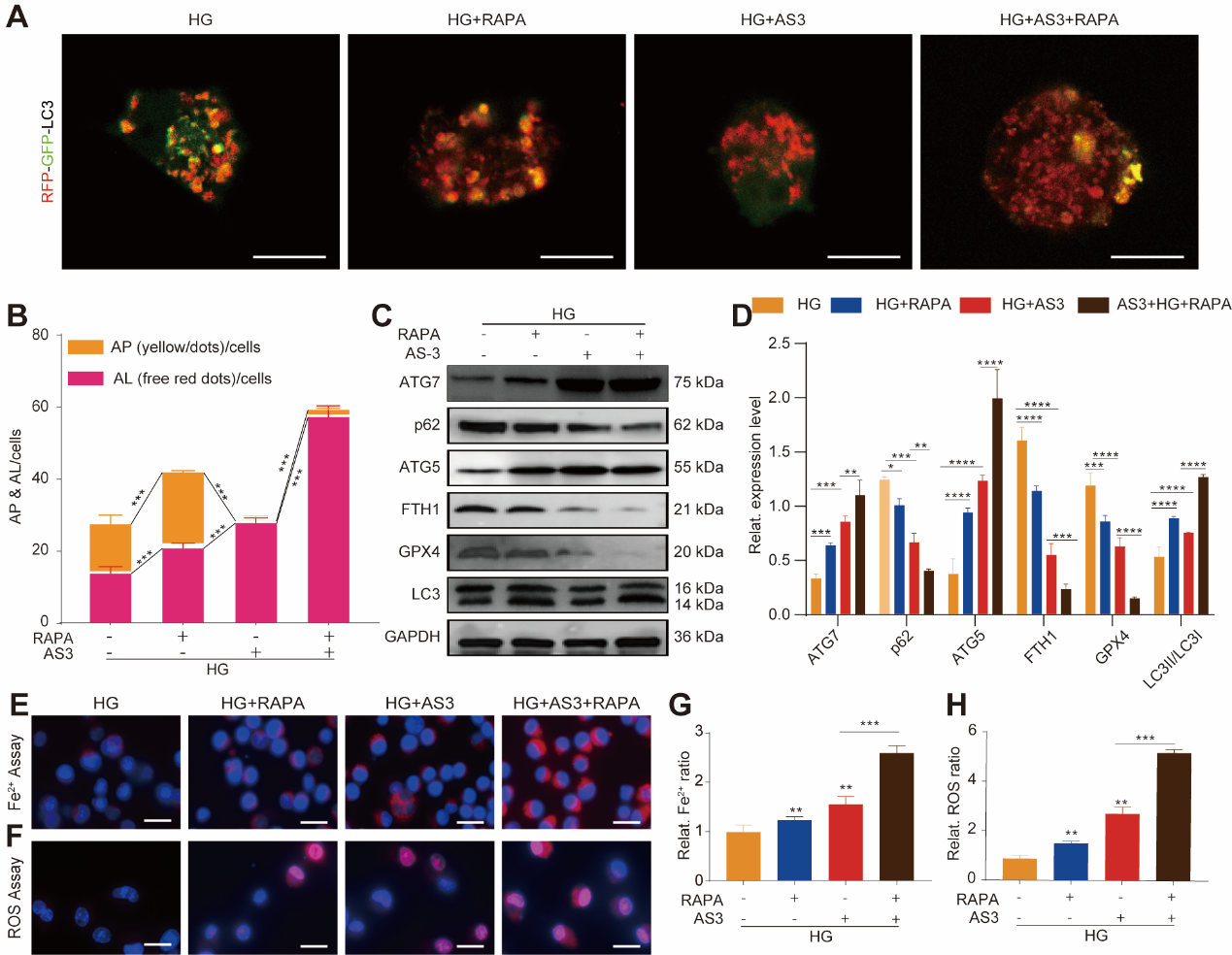


**Figure S13.** The critical roles of autophagy in mediating AS-activated ferroptosis of HG-M1 macrophages. (A) Ad-mRFP-GFP-LC3 double fluorescence indicator experiment showing the activation of autophagy flux in HG-M1 macrophages with different treatments. Scale bar, 10 μm. (B) Quantitative analysis of the mean numbers of APs (indicated by yellow fluorescent dots) and ALs (indicated by red fluorescent dots) in the merged area per HG-M1 macrophages with different treatments (Mean ± SD; one-way ANOVA, n = 3). (C) Western blotting images and (D) quantitative band intensities showing the expression levels of ATG7, p62, ATG5, FTH1, GPX4, and LC3 proteins inside HG-M1 macrophages with different treatments (n = 3). (E) Representing fluorescent images showing Fe^2+^ concentration inside HG-M1 macrophages with above treatments. Scale bar, 5 μm. (F) Representing fluorescent images of ROS level inside HG-M1 macrophages with above treatments. Scale bar, 5 μm. (G) Quantitative analysis of Fe^2+^ concentration in (E) (Mean ± SD; one-way ANOVA, n = 3). (H) Quantitative analysis of ROS level in (F) (Mean ± SD; one-way ANOVA, n = 3). Statistically significant differences between groups are indicated as follows: **p* < 0.05, ***p* < 0.01, ****p* < 0.001,*****p* < 0.0001.

.


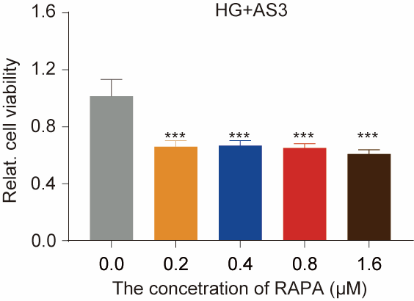


**Figure S14.** CCK-8 assay evaluating the effect of RAPA-pretreatment on cell viability of HG-M1 macrophages with the post-treatment of AS3 (Mean ± SD; one-way ANOVA, n = 6). Statistically significant differences between groups are indicated as follows: ****p* < 0.001.


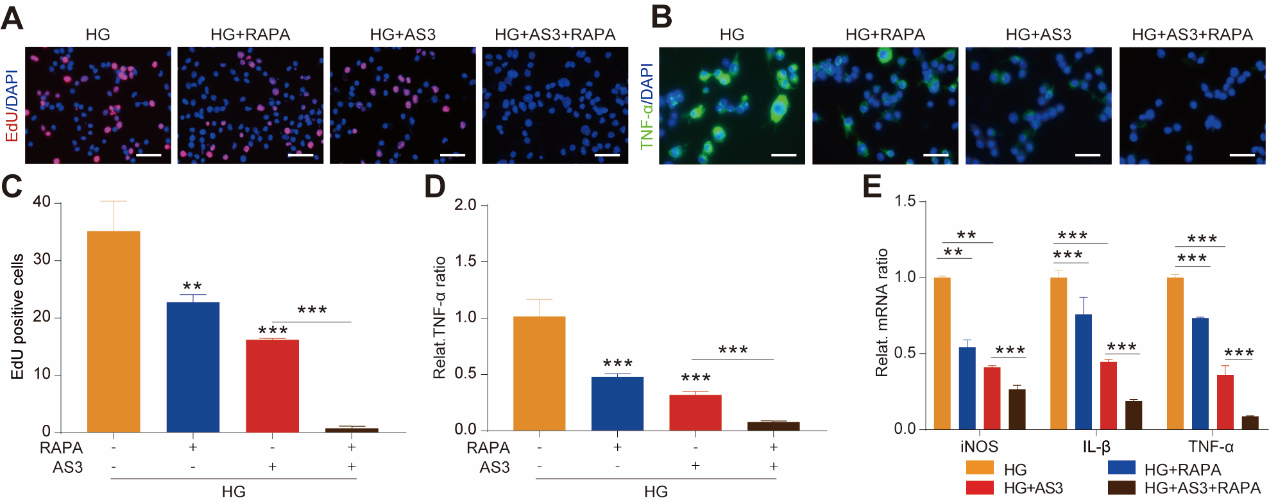


**Figure S15.** The effects of RAPA on the proliferation and secretion capacities of AS-induced HG-M1 macrophages. (A) Fluorescent images of EdU assay (red fluorescence) showing the proliferation behavior of HG-M1 macrophages with above treatments. Scale bar, 100 μm. (B) Fluorescent images reflecting TNF-α expression levels in HG-M1 macrophages with above treatments. Cell nucleus was dyed with DAPI (blue fluorescence). Scale bar, 100 μm. (C) Quantitative result of EdU assay in (A) (Mean ± SD; one-way ANOVA, n = 3). (D) Quantitative analysis reflecting TNF-α expression levels in (B) (Mean ± SD; one-way ANOVA, n = 3). (E) qRT-PCR results demonstrating the gene expression levels of iNOS, TNF-α, and IL-1β inside RAPA pretreated HG-M1 macrophages with or without the post-treatment of AS3 **(**Mean ± SD; one-way ANOVA, n = 3**).** Statistically significant differences between groups are indicated as follows: ***p* < 0.01, ****p* < 0.001.

**
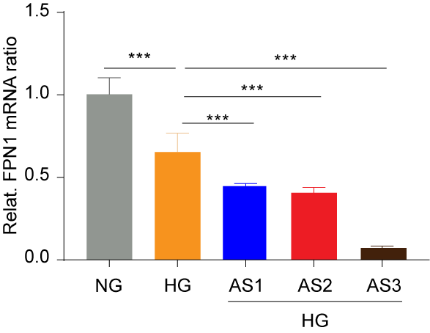
**

**Figure S16.** qRT-PCR results demonstrating the expression level of FPN1 protein inside NG macrophages, HG-M1 macrophages, and AS-treated HG-M1 macrophages **(**Mean ± SD; one-way ANOVA, n = 3**).** Statistically significant differences between groups are indicated as follows: ****p* < 0.001.


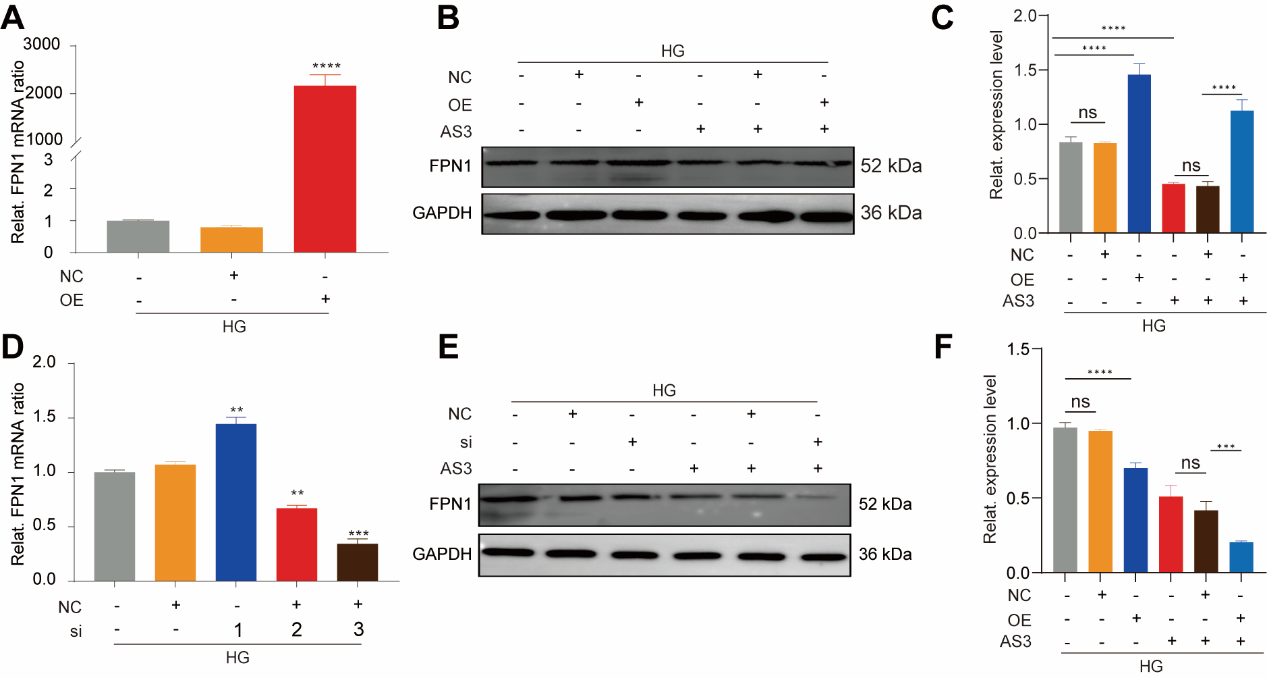


**Figure S17.** The silence or overexpression of FPN1 protein in HG-M1 macrophages was achieved by gene transfection. (A) qRT-PCR results illustrating the expression level of FPN1 gene in HG-M1 macrophages following the transfection with the overexpression plasmid of FPN1 (Mean ± SD; one-way ANOVA, n = 3). Western blotting images (B) and quantitative band intensities (C) showing the expression level of FPN1 protein within HG-M1 macrophages under different treatments (Mean ± SD; one-way ANOVA, n = 3). (D) qRT-PCR results displaying the effectiveness of siFPN1-1, siFPN1-2, and siFPN1-3 in silencing the FPN1 gene inside HG-M1 macrophages (Mean ± SD; one-way ANOVA, n = 3). Western blotting images (E) and quantitative band intensities (F) depicting the expression level of FPN1 protein in HG-M1 macrophages under various treatments (Mean ± SD; one-way ANOVA, n = 3). Statistically significant differences between groups are indicated as follows: ns, not significant, ***p* < 0.01, ****p* < 0.001, *****p* < 0.0001.


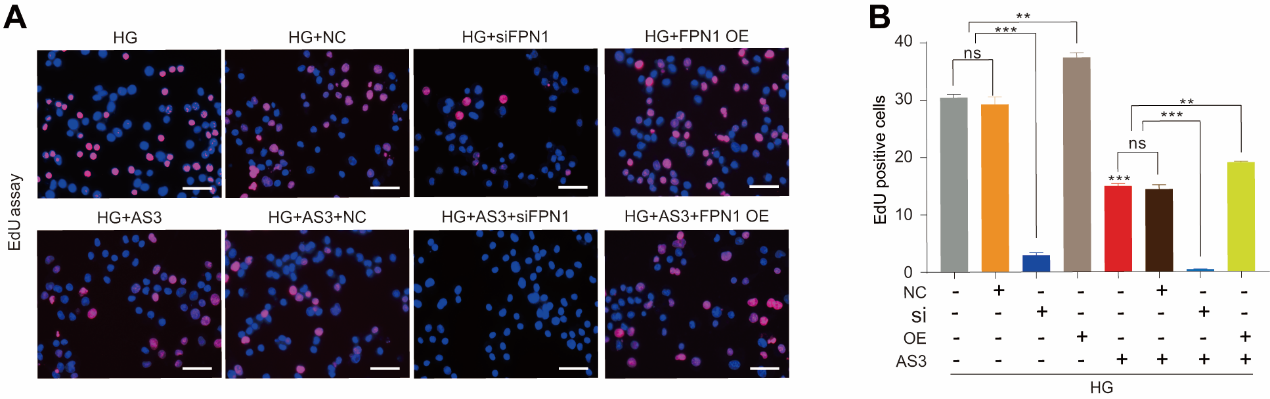


**Figure S18.** (A) Fluorescent images and (B) quantification result of EdU positive cells (red fluorescence) illustrating the effect of FPN1 and the post-treatment of AS on the proliferation behavior of HG-M1 macrophages **(**Mean ± SD; one-way ANOVA, n = 3**).** Scale bar, 50 μm. Cell nucleus was dyed with Hoechst 33342 (blue fluorescence). Statistically significant differences between groups are indicated as follows: ns, not significant, ***p* < 0.01, ****p* < 0.001.


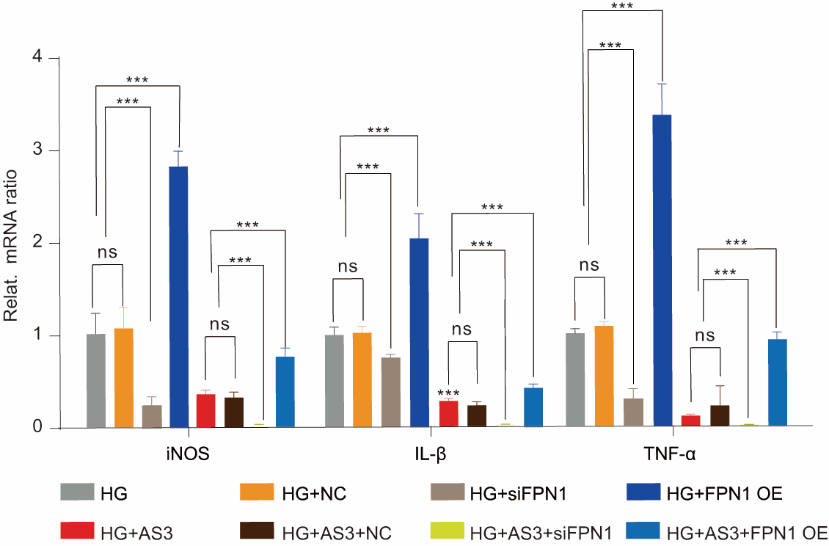


**Figure S19.** qRT-PCR results revealing the expression levels of iNOS, TNF-α, and IL-1β genes within HG-M1 macrophages following different treatments **(**Mean ± SD; one-way ANOVA, n = 3**).** Statistically significant differences between groups are indicated as follows: ns, not significant, ****p* < 0.001.


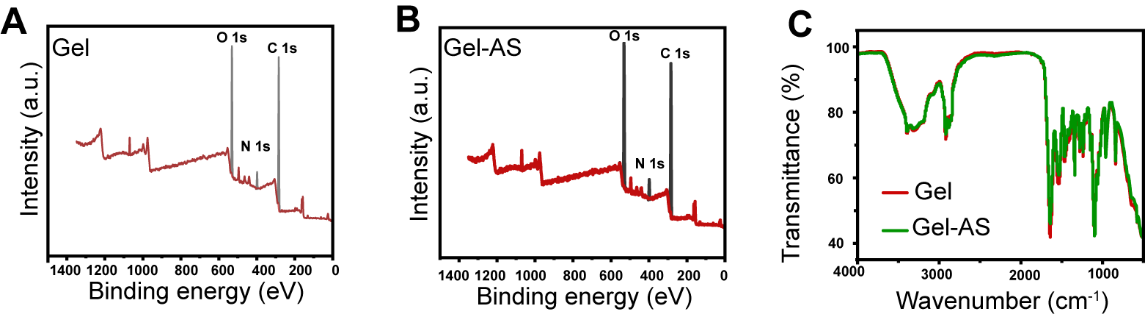


**Figure S20.** XPS and FTIR spectra of Gel and Gel-AS. XPS of Gel (A) and Gel-AS (B). (C) FTIR spectra for Gel and Gel-AS.


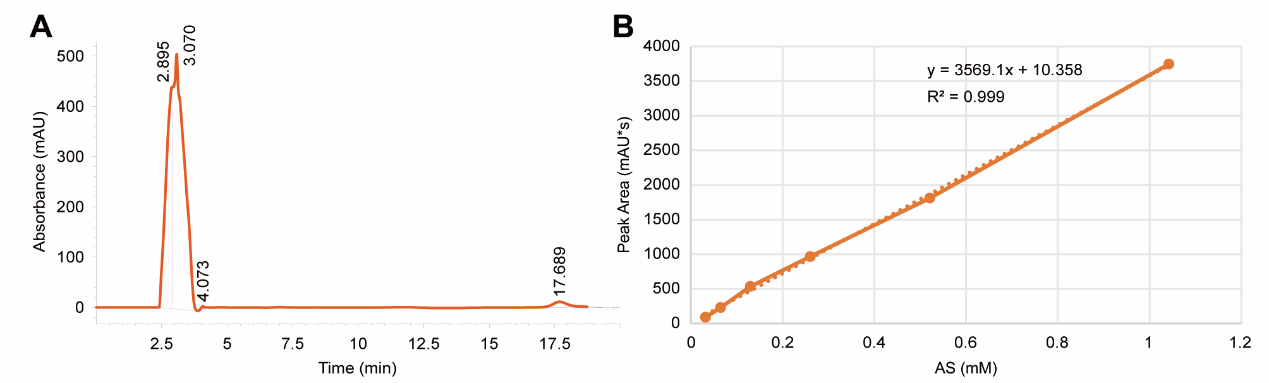


**Figure S21.** Standard curve of AS detected by HPLC. (A) The peak time of AS detected by HPLC. (B) The relationship between peak area and AS concentration is represented by the standard curve.

**
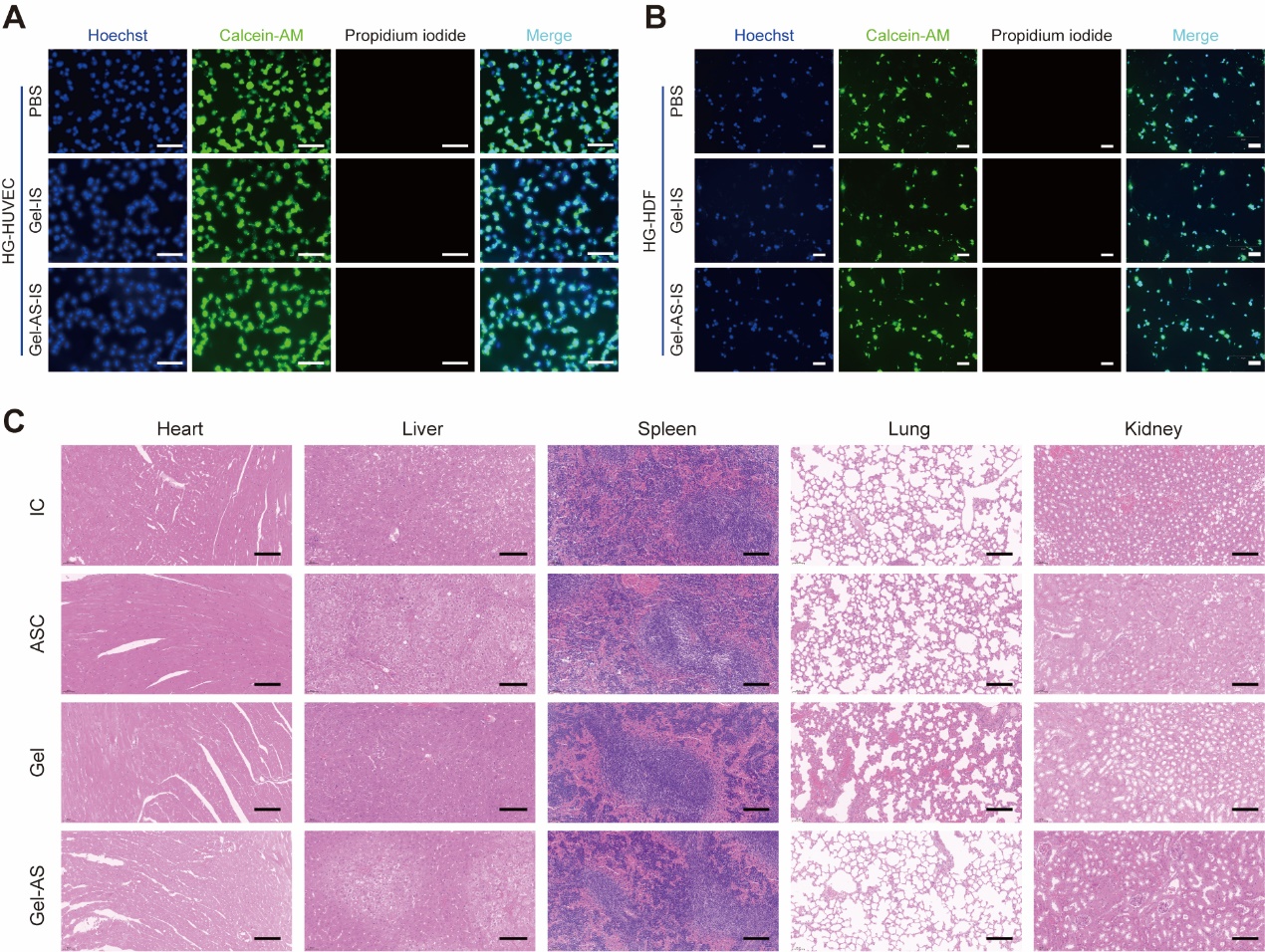
**

**Figure S22.** The *in-vivo* and *in-vitro* biocompatibility of Gel and Gel-AS. Live/dead staining images of HG-HUVECs (A) and HG-HDFs (B) treated with PBS, Gel-IS and Gel-AS-IS for 24 h. Scale bar, 100 μm. (C) H&E staining of major organs in mice treated with IC, ASC, Gel, Gel-AS. Scale bar, 100 μm.


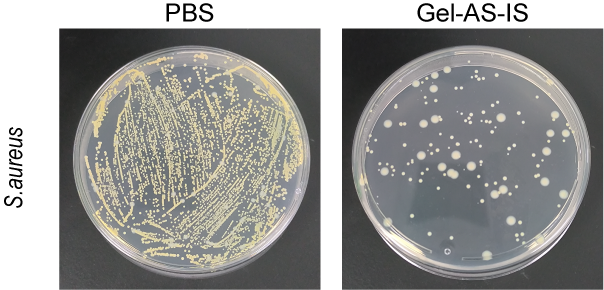


**Figure S23.** The antibacterial ability of Gel-AS-IS against *S. aureus*.


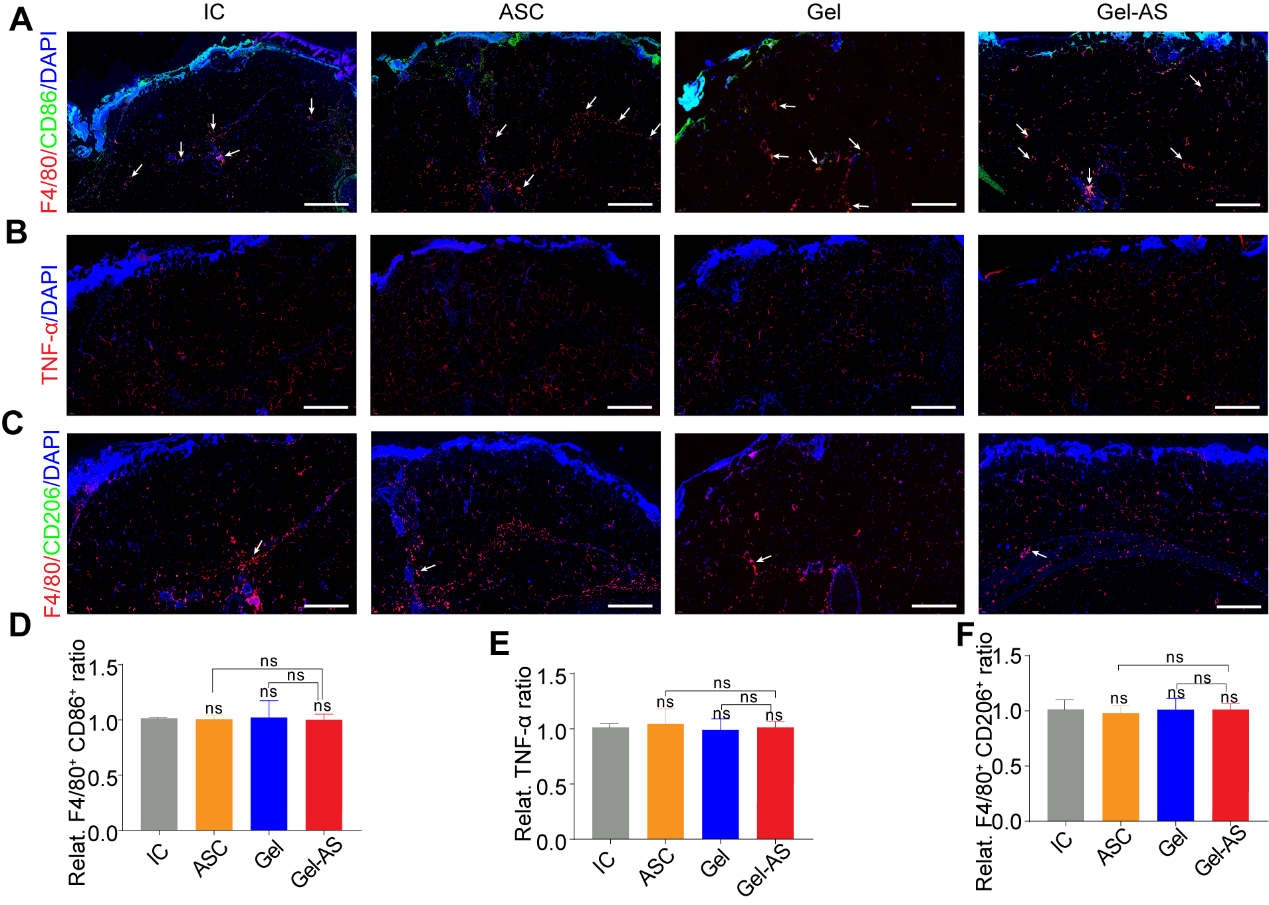


**Figure S24.** AS did not reduce the number of M1 macrophages or reduce the secretion of TNF-α *in vivo* on D2. (A) Immunofluorescent staining images of F4/80^+^CD86^+^ cells (indicated by white arrows) at wounds treated with IC, ASC, Gel, and Gel-AS. Scale bar, 500 μm. (B) Immunofluorescence staining of TNF-α^+^ cells at wounds with different treatments. Scale bar, 500 μm. (C) Immunofluorescent staining images of F4/80^+^CD206^+^ cells (indicated by white arrows) at wounds with different treatments. Cell nucleus was dyed with DAPI (blue fluorescence). Scale bar, 500 μm. (D) Corresponding quantitative statistics analysis of F4/80^+^CD86^+^ cells in (A) (Mean ± SD; one-way ANOVA, n = 6). (E) Quantitative analysis of TNF-α^+^ cells in (B) (Mean ± SD; one-way ANOVA, n = 6). (F) Corresponding quantitative statistics of F4/80^+^CD206^+^ cells in (C) (Mean ± SD; one-way ANOVA, n = 6). Statistically significant differences between groups are indicated as follows: ns, not significant.

Table S1: The primer sequences of genes.

| Genes | sequences |
| --- | --- |
| iNOS-F | ACATCGACCCGTCCACAGTAT |
| iNOS-R | CAGAGGGGTAGGCTTGTCTC |
| IL-1β-F | TTCAGGCAGGCAGTATCACTC |
| IL-1β-R | GAAGGTCCACGGGAAAGACAC |
| TNF-α-F | CAGGCGGTGCCTATGTCTC |
| TNF-α-R | CGATCACCCCGAAGTTCAGTAG |
| ATG5-F | CACCCCTGAAATGGCATTATCC |
| ATG5-R | TGGACAGTGTAGAAGGTCCTTT |
| ATG7-F | TGACCTTCGCGGACCTAAAGA |
| ATG7-R | CCCGGATTAGAGGGATGCTC |
| p62-F | GAACTCGCTATAAGTGCAGTGT |
| p62-R | AGAGAAGCTATCAGAGAGGTGG |
| LC3B-F | TTATAGAGCGATACAAGGGGGAG |
| LC3B-R | CGCCGTCTGATTATCTTGATGAG |
| FTH1-F | TGCCTCCTACGTCTATCTGTC |
| FTH1-R | GTCATCACGGTCTGGTTTCTTT |
| FPN1-F | GGGTGGATAAGAATGCCAGAC |
| FPN1-R | CCTTTGGATTGTGATCGCAGT |
| GAPDH-F | AATGGATTTGGACGCATTGGT |
| GAPDH-R | TTTGCACTGGTACGTGTTGAT |
